# Automated de Novo Design of Olefin Metathesis Catalysts: Computational and Experimental Analysis of a Simple Thermodynamic Design Criterion

**DOI:** 10.1021/acs.jcim.3c01649

**Published:** 2024-01-10

**Authors:** Marco Foscato, Giovanni Occhipinti, Sondre H. Hopen Eliasson, Vidar R. Jensen

**Affiliations:** Department of Chemistry, University of Bergen, Allégaten 41, N-5007 Bergen, Norway

## Abstract

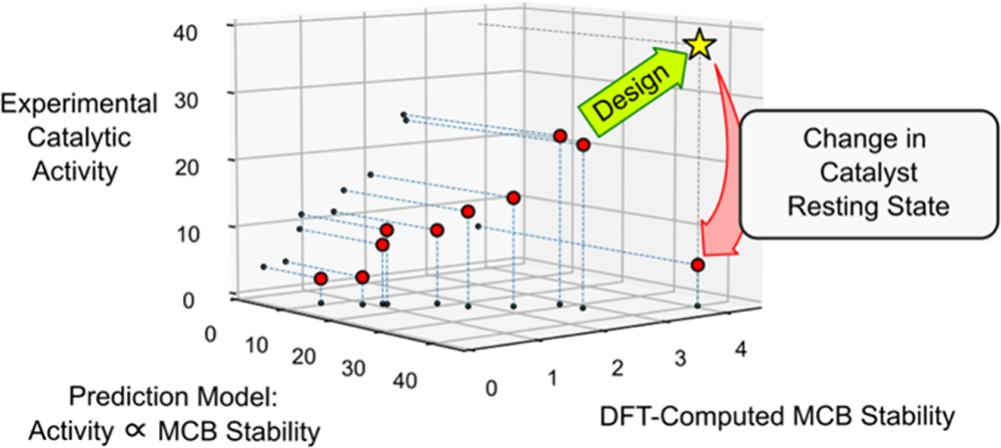

Methods for computational de novo design of inorganic molecules have paved the way for automated design of homogeneous catalysts. Such studies have so far relied on correlation-based prediction models as fitness functions (figures of merit), but the soundness of these approaches has yet to be tested by experimental verification of de novo-designed catalysts. Here, a previously developed criterion for the optimization of dative ligands L in ruthenium-based olefin metathesis catalysts RuCl_2_(L)(L′)(=CHAr), where Ar is an aryl group and L′ is a phosphine ligand dissociating to activate the catalyst, was used in de novo design experiments. These experiments predicted catalysts bearing an N-heterocyclic carbene (L = **9**) substituted by two N-bound mesityls and two *tert*-butyl groups at the imidazolidin-2-ylidene backbone to be promising. Whereas the phosphine-stabilized precursor assumed by the prediction model could not be made, a pyridine-stabilized ruthenium alkylidene complex (**17**) bearing carbene **9** was less active than a known leading pyridine-stabilized Grubbs-type catalyst (**18**, L = H_2_IMes). A density functional theory-based analysis showed that the unsubstituted metallacyclobutane (MCB) intermediate generated in the presence of ethylene is the likely resting state of both **17** and **18**. Whereas the design criterion via its correlation between the stability of the MCB and the rate-determining barrier indeed seeks to stabilize the MCB, it relies on RuCl_2_(L)(L′)(=CH_2_) adducts as resting states. The change in resting state explains the discrepancy between the prediction and the actual performance of catalyst **17**. To avoid such discrepancies and better address the multifaceted challenges of predicting catalytic performance, future de novo catalyst design studies should explore and test design criteria incorporating information from more than a single relative energy or intermediate.

## Introduction

Contemporary design of transition-metal catalysts may draw upon computational molecular design methods that automatically search for catalysts that match a given design criterion optimally.^1^ Such methods, which have decades of history in drug design, are finally being developed for and adopted in the design of molecules beyond drug-like organic molecules, including design of transition-metal catalysts.^2,3^ Opening these new possibilities has required the development and adaptation of cheminformatics for the handling of transition-metal and organometallic chemistry, such as that of automated construction of models of catalytic intermediates and transition states.^4−8^

However, these cheminformatics tools must be complemented by a design criterion.^1^ While catalytic systems are inherently complex,^9^ a simple and tempting starting point for developing a catalysis-relevant criterion is offered by Sabatier’s principle: An effective catalyst should interact not too weakly and not too strongly with the substrate.^10−12^ In homogeneous catalysis, this thermodynamic principle has, when extended with kinetic factors,^13^ recently been proposed as an approach with which to evaluate homogeneous catalysts in terms of volcano plots,^14−17^ which are tools commonly used in heterogeneous catalysis.^12,18,19^

The power of volcano plots as catalyst evaluation tools lies in the condensing of the energetics of a catalytic cycle into very intuitive pictures.^17^ In a conceptually similar complexity-reducing strategy, the energetic span model,^20−23^ the estimated turnover frequency (TOF) is expressed in terms of the intuitively attractive energy span between TOF-determining intermediates and transition states.

Even if highly useful, complexity-reducing tools for evaluating catalytic activity rely on the free-energy profiles of the relevant catalytic cycles, the calculation of which is costly. Fortunately, the costs may be reduced by exploiting correlations between the stability of various intermediates and transition states, such as in linear free-energy scaling relationships (LFESRs),^24−26^ in Hammett’s equation, and in the Bell–Evans–Polanyi model.^27−29^ Using such relations, a single calculated relative energy or even a structure–activity relationship based on a few calculated molecular descriptors^30,31^ may provide estimates of the stability of further intermediates and transition states of the catalytic cycle. The more broadly the underlying assumptions and correlations are valid across chemical space, the fewer the calculations that will have to be made to evaluate candidate catalysts.

A critical question thus is to what extent such correlations can be relied upon in catalyst design. Whereas linear scaling relationships are routinely exploited in heterogeneous catalysis, the variability resulting from through-space interactions (both electronic and steric) and geometrical distortions induced by structurally different ligands in homogeneous catalysis is larger than that normally seen in heterogeneous catalysis.^17,32,33^ In fact, the breaking of linear scaling relationships in molecular systems appears to be common and has even been exploited to gain mechanistic understanding^34^ or to identify changes in the mechanism between otherwise similar catalysts.^35^ Moreover, differences in scaling relations have been observed for the same reaction mediated by catalysts bearing ligands of different kinds.^13^ Needless to say, prediction models based on such context-specific relations are applicable only in a very limited portion of the chemical space.

Such limitations may quickly become detrimental in automated de novo design, the very nature of which is to go beyond known structures. De novo methods, rather than screening known and similar candidates, relentlessly move toward optimal solutions. To the observer, the corresponding routes taken in chemical space will often appear to be based on extrapolation rather than interpolation between known candidates. In other words, automated de novo methods by their very nature can be expected to stress-test prediction models and push their boundaries. However, even if expected, the degree to which this problem actually limits the use of correlation-based strategies in de novo catalyst design remains unexplored.

In this contribution, we explore, for the first time, whether a presumably solidly determined correlation-based catalyst design criterion derived from a quantitative structure–activity relationship (QSAR) study of a broad range of relevant catalysts is valid for de novo-designed catalysts. These explorations include detailed mechanistic calculations covering portions of the catalytic cycle far beyond that of the initial correlation and actual experimental follow-up and validation. To our knowledge, an experimental follow-up of newly designed homogeneous transition-metal catalysts has been performed only for designs resulting from virtual screening,^36,37^ that is, for explorations within a landscape of known compounds, but never for de novo-designed candidates.

**Scheme 1:**
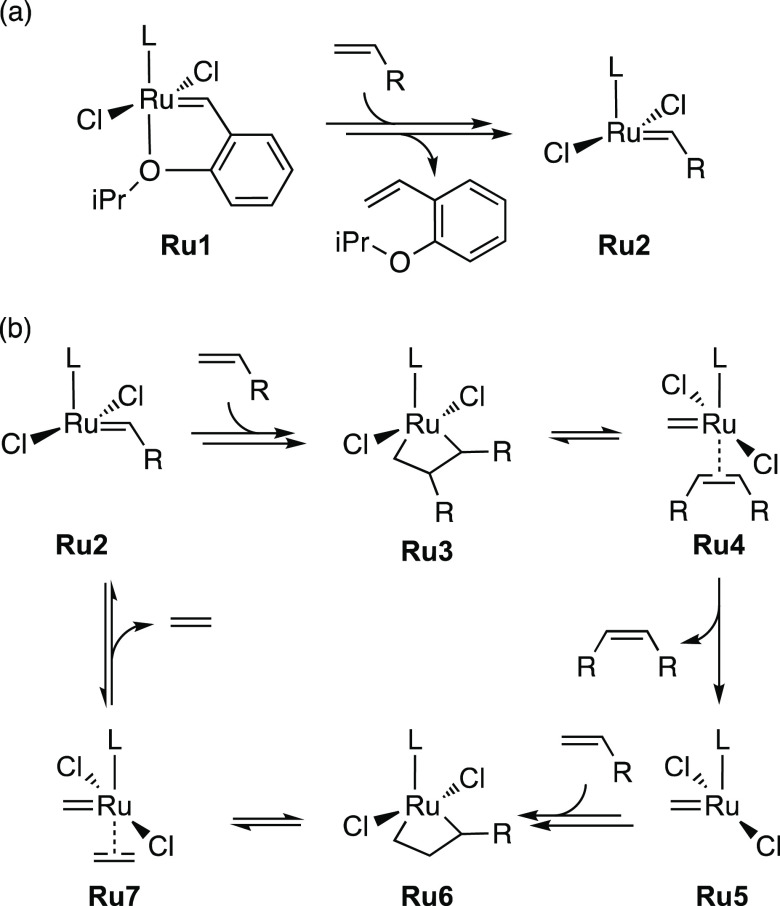
Simplified Reaction Mechanism of Ruthenium-Catalyzed Olefin Metathesis: Precatalyst Initiation (a) and Self-Metathesis of Terminal Olefins (b)

The catalytic reaction chosen for the present de novo explorations is that of ruthenium-catalyzed olefin metathesis (Scheme 1). Olefin metathesis is the most versatile and atom-economic method with which to form carbon–carbon double bonds.^38,39^ Due to this versatility and the robustness and ease with which the ruthenium catalysts can be used, ruthenium-catalyzed olefin metathesis holds great promise as a green technology for pharmaceutical manufacturing,^40,41^ valorization of renewable feedstocks,^42,43^ and materials science.^44−46^

**Chart 1:**
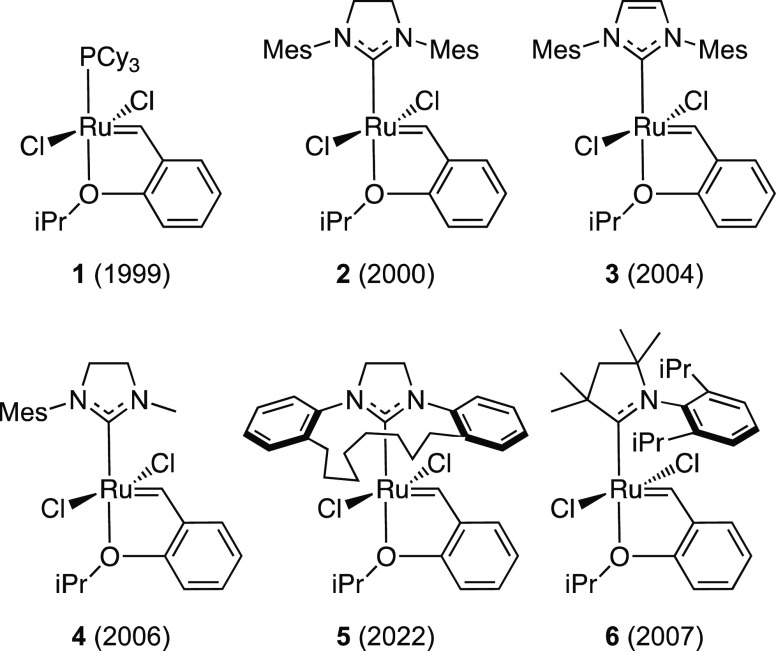
Selected Ruthenium-Based Olefin Metathesis Catalyst Precursorsa^,^b

To deliver on the promise and foster industrial uptake of ruthenium-based olefin metathesis catalysts,^41−43^ improvements within the catalyst precursor paradigm RuX_2_(L)(L′)(=CHAr) (X = anionic ligand, L, L′ = dative ligand, and Ar = aryl group) developed by Grubbs et al.^47−49^ are continuously being sought. A stability-promoting improvement has been the tethering of a labile dative ligand via the alkylidene achieved in the Hoveyda-class catalyst precursors RuCl_2_(L)(=CHAr) (Ar = C_6_H_4_-2-O^*i*^Pr) in which the isopropoxy group dissociates to initiate catalysis (Chart 1).^50,51^ Also seen in Chart 1 are some of the many variations of the remaining donor ligand L. This ligand has been the subject of numerous modifications and attempts at improving the activity, stability, and other catalytic properties. Carbene-type ligands, in particular, offer almost unlimited possibilities for structural variation, generating a search space ideally suited for exploration by de novo design strategies.

a The year of publication is reported in parentheses for catalysts **1**,^50^**2**,^52^**3**,^53^**4**,^54^**5**,^55^ and **6**.^56^ b Mes = 2,4,6-trimethylphenyl.

**Scheme 2:**
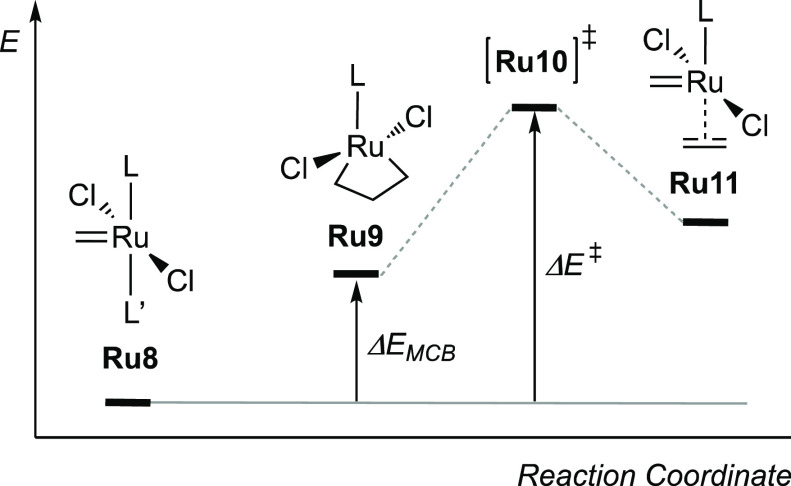
Definition of Energy Descriptors along the Ethylene Self-Metathesis Reaction

In addition to presenting a suitable challenge for molecular design, ruthenium-catalyzed olefin metathesis offers intermediates already shown to provide correlations useful for catalyst design.^31^ First, the overall energy barrier to ethylene self-metathesis (Δ*E*^‡^ in Scheme 2), the simplest possible olefin metathesis model reaction, has been shown to correlate strongly (*R*^2^ = 0.985; Figure S1) with the relative stability of the metallacyclobutane (MCB) intermediate **Ru9** (Δ*E*_MCB_ in Scheme 2).^57^ The latter stability in turn was found to correlate with molecular descriptors of methylidene complex **Ru5**.^31^ Thus, calculating the properties of this relatively small 14-electron complex suffices for estimating via QSAR models, the stability of the MCB intermediate, and thus also, via the correlation between Δ*E*_*MCB*_ and Δ*E*^‡^, the barrier to ethylene self-metathesis. By far, the most important descriptor of the final QSAR model was the length of the Ru=CH_2_ bond in **Ru5**, which correlated inversely with catalyst productivity (*R*^2^ = 0.859; Figure S2).

This simple interatomic distance-based correlation is the design criterion tested in the present work. To minimize the influence of inaccurate molecular modeling, a relatively costly (in terms of computational time) and presumably accurate electronic structure method based on density functional theory (DFT) is used to obtain the geometry and Ru=CH_2_ bond distance of **Ru5**. In other words, although the design criterion relies on two correlations, the key molecular descriptor is expected to be accurately determined. With this criterion, de novo artificial evolution experiments are performed to automatically design N-heterocyclic carbene (NHC) ligands (L) that in theory should promote productive olefin metathesis. The soundness of this approach is assessed by additional mechanistic DFT calculations and experimental follow-up.

## Methods

### De Novo Design of NHC Ligands

De novo design experiments were performed using the open-source software package De Novo OPTimization of In/organic Molecules (DENOPTIM) for fully automated in silico molecular design.^2^ Using a genetic algorithm in which the fitness or figure of merit of each candidate L ligand was given by the negative of the DFT-calculated Ru=CH_2_ bond distance of **Ru5**, that is, –*r*(Ru=CH_2_), DENOPTIM designed NHC ligands (L) automatically by seeking to maximize the fitness. The density functional approximation, basis sets, and other specifics of the molecular modeling protocol (here referred to as Prediction Model 1) used in the de novo experiments are described in Section S2.1.1.

In each evolutionary experiment, a randomly generated initial population of candidate ligands was iteratively evolved by subjecting some of the good candidates to genetic operations, such as mutation (modification of a portion of the molecular structure of a candidate ligand) and crossover (two offspring molecules created by exchanging portions of the molecular structures of two parent molecules). Further details of the genetic algorithm are described in refs (58) and (8). Moreover, all data and tools needed to reproduce the present results are available via the Zenodo^59^ Open-Science database.^60^

**Chart 2:**
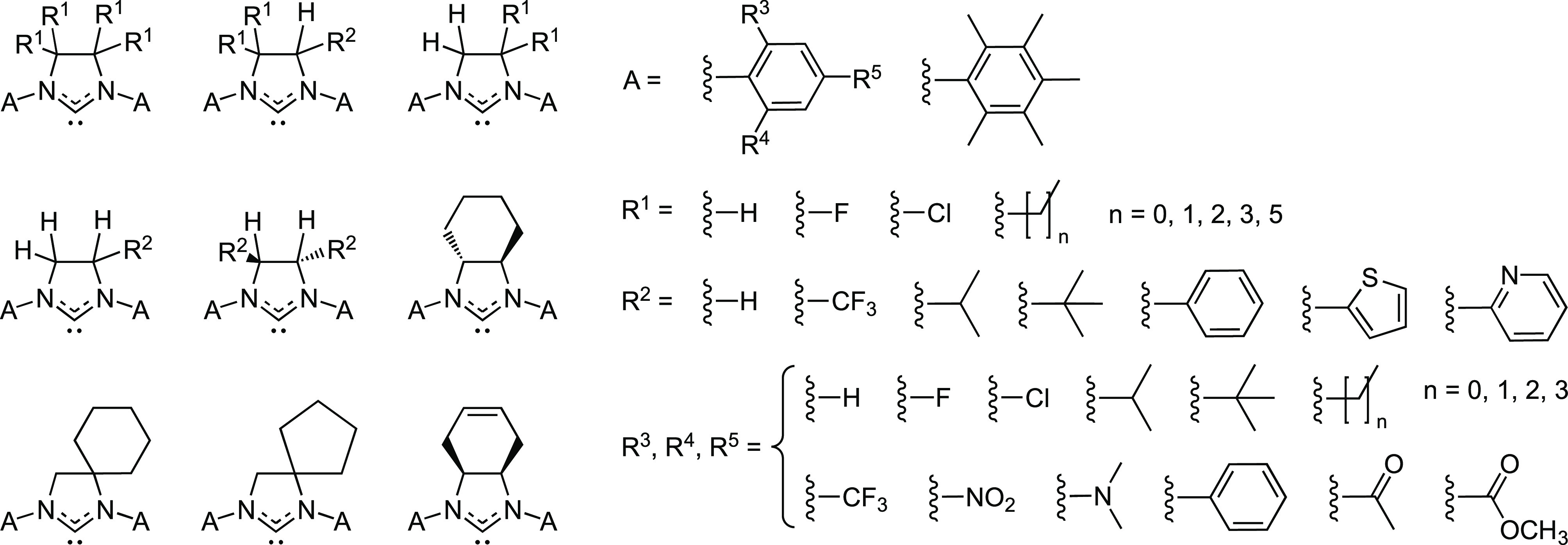
Fragment Space Used in the De Novo Design Experiments

The generation and modification of NHC ligands were controlled by a predefined fragment space: a collection of molecular building blocks and rules for combining them into molecules.^6^ The fragment space was designed to explore electronic and steric effects and foster synthesizability, for example, by avoiding Bro̷nsted-acidic groups (e.g., OH) that could trigger catalyst decomposition. The fragment space is illustrated in Chart 2. The NHC ligands of the fragment space are characterized by (i) the imidazolidin-2-ylidene scaffold, (ii) aromatic N-substituents (A in Chart 2, required to be the same on both N atoms of an NHC ligand), and (iii) the optional functionalization of the carbon atoms of the NHC backbone. As seen in Chart 2, a degree of symmetry is required for the backbone substituents R^1^ and R^2^.

The fragment space encodes 154 882 unique ligand constitutions. However, since substituents at the NHC backbone may prevent twisting around the N–aryl bond,^61^ asymmetric aryl groups A (R^3^ ≠ R^4^ in Chart 2) can, in some cases, offer experimentally isolable conformers.^62−64^ To account for this possibility, we represent such conformers as independent species^65^ even though this leads to the generation of a few duplicate candidates. In other words, conformers of NHC ligands in which twisting around either of the N–A bonds is energetically accessible at room temperature are here treated as independent candidates (i.e., configurations) even if they are indistinguishable in standard experiments.

### Computational Validation of de Novo-Designed Ligands

To verify the accuracy of the *r*(Ru=CH_2_)-based correlation, we first considered its sensitivity to the approximations implemented in the modeling of the Ru=CH_2_ bond by Prediction Model 1. Notably, the correlation coefficient between Ru=CH_2_ bond distances produced by modifications of Prediction Model 1 and the thermodynamic stability of **Ru9** remains high (R^2^ > 0.92) even when extended basis sets and/or an implicit solvation model are used (Section S1.3).

Next, the average (over L′ = PMe_3_, H_2_O, and CH_2_O)^31^ enthalpic difference between the **Ru9** and **Ru8** was calculated for the fittest candidates produced by the artificial evolution experiments. The average includes a model of a ruthenium–methylidene phosphine adduct (L′ = PMe_3_ as a model phosphine), an adduct with a catalyst poison (L’ = H_2_O), and an adduct with a carbonyl-containing substrate (L′ = CH_2_O as a model carbonyl),^31^ and the corresponding average enthalpic difference has been found to vary qualitative with the experimentally determined catalytic activity and has been used as a simple, surrogate measure of catalytic productivity.^31^ The computational model used here to calculate this productivity is termed Prediction Model 2 (see Section S2.1.2).

Whereas the above-described computational models used in de novo design (Prediction Model 1) and fast computational validation of predicted catalysts (Prediction Model 2) were created for efficiency, a third computational model (Reactivity Model, see Section S2.1.3) was created to investigate in detail the correspondence between the computationally predicted and the experimental results. The latter model was created for accuracy and consistency with prior mechanistic work on ruthenium-based olefin metathesis.^66,67^

### Experimental Validation

The most promising candidate catalyst remaining after the de novo design and the subsequent computational validation (the calculation of the productivity) was synthesized, characterized, and experimentally tested for catalytic performance. See Section S3 for details of the experimental methods used.

## Results and Discussion

### De Novo Design

**Figure 1:**
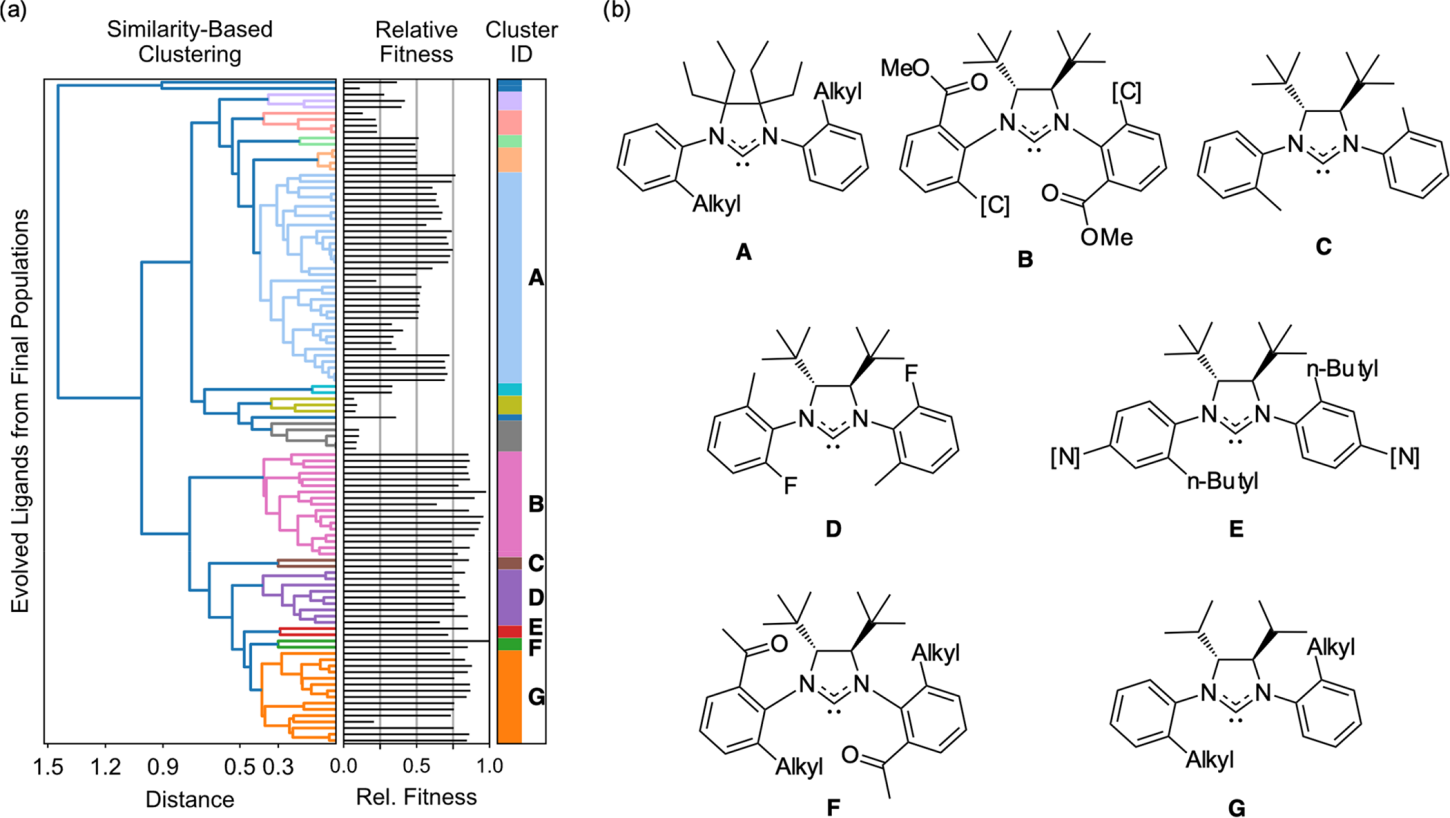
Analysis of the best candidates identified by the evolutionary experiments (the Fittest Set; see text): (a) dendrogram organizing the ligands of the Fittest Set according to chemical similarity (i.e., Tanimoto similarity of *rdkit*(68) fingerprints). The normalized relative fitness of each candidate is shown in the aligned histogram. Dendrogram’s branches are colored so as to facilitate the identification of clusters. Clusters mentioned in the text are identified by “Cluster ID” labels **A**–**G**. (b) Maximum common substructure for candidates in clusters **A**–**G**. Label [C] represents a carbon-based substituent that is either an alkyl or a carbonyl-based group (acetyl or ester). Label [N] represents either a nitro or dimethylamino substituent. The identity of each candidate ligand is available in the repository of this contribution.^60^

Seven evolutionary de novo design experiments were performed, which required computational evaluation (fitness calculation) of 1435 ligands in total. To identify the chemical features contributing to a high fitness, the fittest candidates from each evolutionary experiment were collected in a single set, here referred to as the *Fittest Set*, containing 107 ligands. Since independent evolution experiments converged to ligands with similar chemical features, a cluster analysis based on the Tanimoto similarity using the candidate’s *rdkit*(68) fingerprints was performed. The maximum common substructure (MCS)^69^ within each cluster is highlighted as shown in Figure 1. Whereas clusters were not identified considering fitness values, cluster members tend to have similar fitness and thus indirectly serve to identify the structure–property relationship.

The maximum common substructures of the high-fitness clusters (Figure 1) reveal two structural features that help shortening the Ru=CH_2_ bond and increasing the productivity: (a) sterically demanding alkyl substitution on the backbone of the imidazolidin-2-ylidene and (b) *N*-aryl groups with alkyl and/or carbonyl-containing substituents (ester or acetyl) in the *ortho* positions.

The first structural feature (a) is typically achieved by having one *tert-*butyl group on each carbon atom of the backbone (cluster represented by MCS **B**–**F** in Figure 1) or via a tetra-alkyl-substituted backbone (cluster **A**). However, cluster **A** is outperformed by clusters **B**–**G**. Among the latter, **B** and **F** are characterized by a carbonyl-based group (acetyl, ester) in at least one of the *ortho* positions of the *N*-aryl group. Since **B** and **F** contain four and one of the five best candidates, respectively, this structural feature at first glance appears to be a booster for high fitness. Notably, this structural feature is not prominent among known highly active catalysts. In contrast, clusters **C**–**G** contain ligands featuring at least one alkyl substituent at the N-aryl *ortho* positions, which is consistent with the abundance of such substituents among existing highly active catalysts for olefin metathesis.

When attempting to rationalize why the above structural features dominate among the fittest candidates, we note that alkyl substituents on the NHC backbone increase the ligand-to-metal electron donation of the NHC ligands,^31,70^ which has been found to strengthen and thus shorten Ru–alkylidene bonds.^31^ In addition to this electronic effect, substitution at the NHC backbone increases the steric repulsion between these substituents and the *N*-aryl ones and thus indirectly also between the NHC and the RuCl_2_(=CHR) fragment. This repulsion leads to longer Ru–NHC bonds and shorter Ru=CH_2_ bonds. The latter steric effect will, of course, be greater the more heavily the *N*-aryl groups are substituted.

Still, even if contributing to activity-promoting steric effects, the high fitness brought about by ester and acyl N-aryl substituents (clusters **B** and **F**) is surprising given that the electron-withdrawing effect of these substituents should lower the donating capabilities of the NHC and thereby the catalytic activity. For now, we note that, hypothetically, this could indicate that the electronic effect of substitution at the *ortho* positions is small compared to the steric effect. The effects of these substituents and other structural features are analyzed in the following.

**Chart 3:**
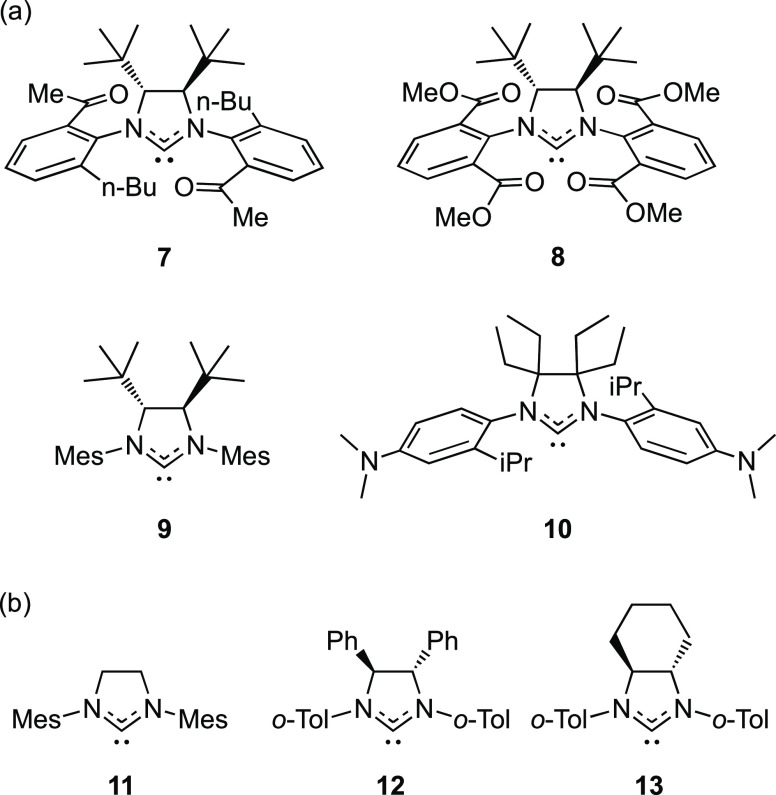
(a) Representative Members of the Fittest Set of Automatically Designed Ligands and (b) Selected NHC Ligands of Existing Catalystsa

To evaluate the catalyst properties without relying on the correlation between the length of the Ru=CH_2_ bond and the stability of the MCB intermediate, we calculated the latter directly (using Prediction Model 2) for a handful of predicted (Chart 3a) and known (Chart 3b) ligands, resulting in the productivities listed in Table 1. First, we note that the carbonyl groups of **7** and **8** increase the stability of **Ru8** by forming ancillary bonds with the metal and metal-coordinated dative ligands L′ (Figure S9). However, such interactions were not part of the training set of Prediction Model 2.^31^ With carbonyl groups being incompatible with Prediction Model 2, we cannot confirm the catalytic potential of ligands containing such groups. Hence, we discard ligands **7** and **8** and turn our attention to ligands **9** and **10**. The catalytic potential predicted via the Ru=CH_2_ bond distance for the latter ligands is corroborated by their directly predicted productivities (Table 1). Given the reported activity-reducing effects of combining high degrees of both NHC backbone and N-aryl substitution,^70^ ligand **10**, featuring a tetra-ethyl-substituted backbone (a challenge for synthesis) as well as a bulky N-aryl substituent (iPr), appears to be unattractive for an experimental follow-up study. Instead, ligand **9** might offer activity-promoting ligand-to-metal donation without exaggerating the overall degree of substitution.

a Mes = 2,4,6-trimethylphenyl. *n*-Bu = *n*-Butyl. *o*-Tol = 2-methylphenyl.

**Table 1:** Comparison of Modeled Ru–Methylidene Bond Distance and Predicted Catalyst Productivity of Known and New Ligands

ligand	Ru=CH_2_ (Å)	productivity
7	1.812	ND^a^
8	1.813	ND^a^
9	1.813	5.4
10	1.813	8.1
11^b^	1.816^c^	2.6^c^
12^b^	1.816^c^	3.7^c^
13^b^	1.817^c^	2.8^c^

a Not determinable.

b Existing catalysts.

c From ref (31).

In fact, the imidazolinium chloride salt of carbene **9** can be prepared via a known procedure.^71^ Despite having been known for approximately a decade, only a single metal complex of **9**, based on silver,^71,72^ has been reported so far. Still, when evaluating the potential of **9** as a ligand in general and in Ru-based olefin metathesis catalysts in particular, it should be kept in mind that NHC ligands featuring two *tert-*butyl substituents at the backbone are not uncommon in organometallic chemistry and are key to a class of highly active and enantioselective ruthenium metathesis catalyst.^73−76^ The ligands of the latter class differ from **9** by one of the NHC nitrogen atoms bearing a relatively small substituent such as methyl, benzyl, or propyl.^73−75^

In conclusion, both the automated de novo design procedure and the subsequent manual computational validation suggest that ruthenium catalysts based on carbene **9** may have potential in olefin metathesis. The available experimental information, including a synthetic procedure for an imidazolinium salt of **9**, also suggests that an experimental follow-up project to check this catalytic potential should be both possible and worthwhile.

### Experimental Verification

With the goal to access a ruthenium alkylidene complex based on the NHC ligand **9**, we followed the most common strategy, which consists of reacting the free carbene with a phosphine-based alkylidene precursor.^77^ The free carbene **9** was prepared by deprotonation of its imidazolinium chloride salt with potassium bis(trimethylsilyl)amide (KHMDS); see Section S3.2 for details. The imidazolinium chloride salt of **9** was purchased from a custom synthesis company (Santai Labs, Inc.) and used as received.

**Scheme 3:**
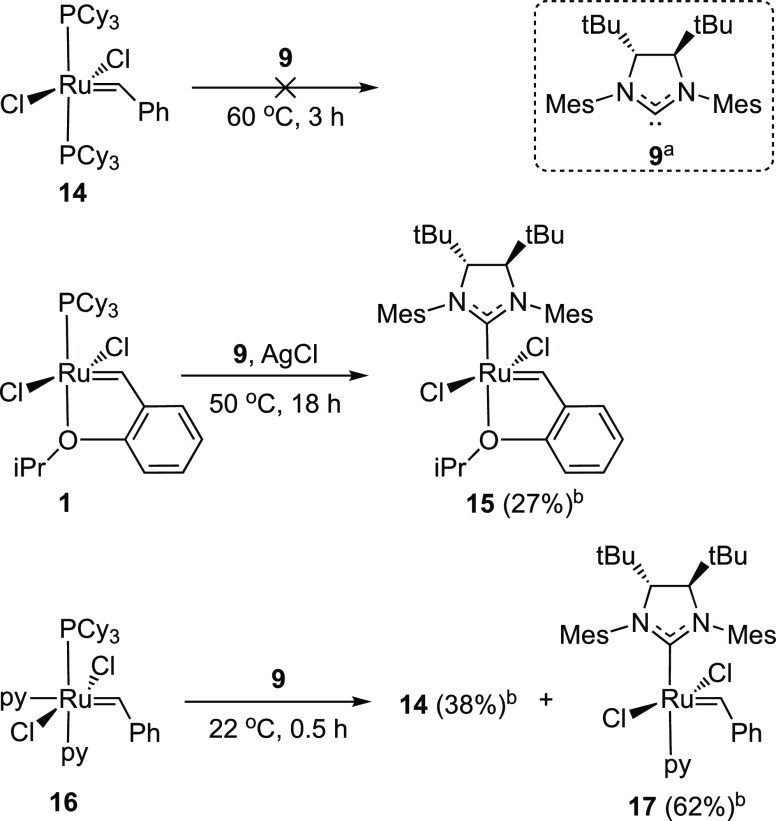
Reaction of Carbene Ligand **9** with Ruthenium Alkylidene Precursors^,^ The free carbene **9** was prepared in situ by heating a mixture of **9**·HCl with potassium bis(trimethylsilyl)amide (KHMDS) at 70 °C for 5 min. Determined by ^1^H NMR of the reaction mixture in C_6_D_6_.

Scheme 3 summarizes the main attempts to install carbene **9** on an alkylidene precursor. The reaction with Grubbs first-generation catalyst **14** did not result in new alkylidene complexes (Scheme 3, top). We repeated the reaction under various conditions, but carbene **9** appeared to be unable to replace the phosphine ligands of **14**. In contrast, the reaction with the Hoveyda-Grubbs first-generation catalyst proceeded, albeit slowly, at 50 °C in the presence of a phosphine scavenger (AgCl, Scheme 3, middle). A new singlet peak at 17.39 ppm (C_6_D_6_) next to the alkylidene resonance of the precursor **1** (17.37 ppm (d, *J* = 4.8 Hz) appeared in the ^1^H NMR spectrum of the reaction mixture after 2 h. At this time, the yield of the new compound, assumed to be **15**, was estimated to be 10%. After 18 h, the yield had increased to 27%, but the presence of decomposition products was also evident. Isolation of the new compound from this mixture was deemed challenging, and we decided to also test the bis-pyridine precursor **16** (Scheme 3, bottom). The reaction was, in this case, fast, and after 30 min at room temperature, the starting complex was entirely converted into a mixture of the target complex **17** (62% by ^1^H NMR) and **14** (38% by ^1^H NMR). After partial removal of the solvent, **17** was precipitated upon the addition of pentane and isolated by vacuum filtration as a green solid (48% yield). The compound was characterized by ^1^H and ^13^C NMR and elemental analysis; see Section S3.2 for details. Attempts to grow crystals suitable for X-ray diffraction analysis failed due to the poor stability of the complex in solution.

**Figure 2:**
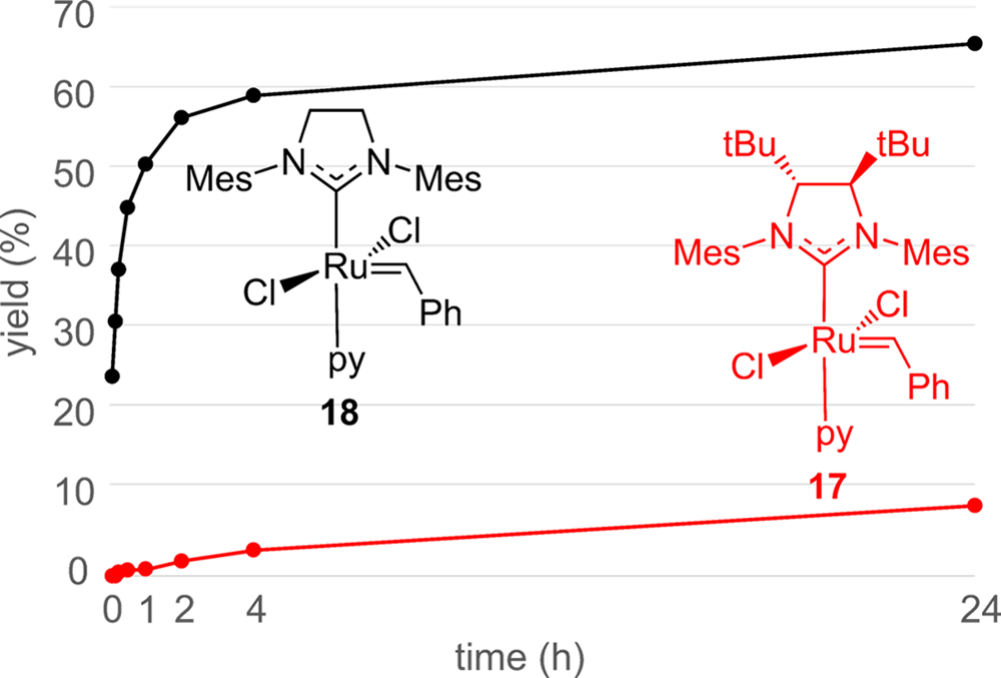
Self-metathesis of neat 1-octene at room temperature with 100 ppm of the catalyst; see Section S3.3 for details. The yields were determined by quantitative ^1^H NMR.

The performance of **17** as a catalyst for 1-octene self-metathesis was compared with that of the well-known and very similar Grubbs third-generation catalyst **18** (Figure 2). **18** initiates considerably faster and is also more productive than **17**. To illustrate, whereas 100 ppm of **18** gives 25% of self-metathesis product in neat 1-octene after only 5 min, **17** produces only traces of the product (<1%) during the first hour. These product yields reach 67% for **18** and only 9% for **17** after 24 h. For both **17** and **18**, catalyst initiation appears to correlate with their solubility in the 1-octene substrate: **17** and **18** are essentially insoluble in nonpolar media such as pentane or hexane, and their dissolution in 1-octene is likely to proceed via alkene coordination to the ruthenium center and catalyst initiation. This associative mechanism is available for **18**(78) and is assumed to operate for both **17** and **18** under the neat-substrate conditions used here. The greater steric bulk of **9** should be expected to retard alkene binding for **17** versus **18**, which is consistent with the slower observed disappearance of the former solid: Whereas solid **18** disappears within minutes in 1-octene, **17** requires ca. 4 h. Thus, the observed slower disappearance and lower yields of **17** early in the experiment are both indications of a slower initiation. However, at 24 h, both solid precursors have been absent for most of the experiment, and the product yields, clearly in favor of the established catalyst **18**, are little influenced by the initiation.

To reduce the influence of the initiation rate and catalyst solubility even further from the comparison, we repeated the self-metathesis reaction at 60 °C with 1 ppm of the catalyst. Under these conditions, both catalysts were completely dissolved in the substrate at the start of the reaction. After 1 h, the yield of the self-metathesis product was 49% (TON = 245 000) for **18** versus 12% (TON = 60 000) for **17** with the yield of isomerization products, which are a sign of catalyst decomposition,^79^ being 4% (**18**) and 13% (**17**).

Overall, these results show that, in contrast to the prediction offered by the distance-based design criterion, **17** is a significantly less productive and efficient catalyst than **18**, and it also initiates more slowly. The larger fraction of isomerization products also suggests that **17** is less stable than **18**. The lower stability of **17** and the difficulties encountered when installing carbene **9** on ruthenium alkylidenes suggest that **9** forms weaker bonds with ruthenium than carbene **11**, which is the key dative ligand in leading catalysts such as Hoveyda-Grubbs second-generation catalyst (**2**) and Grubbs third-generation catalyst (**18**). To test this hypothesis and, more generally, offer insight into the discrepancy between the computationally predicted and experimental results, we next set out to investigate using DFT calculations carbene ligands **9** and **11** and their corresponding catalysts **17** and **18** in more detail.

### Mechanistic DFT Investigation

Calculations to uncover the origin of the discrepancy between the predicted and the experimental results were performed using a computational model (termed Reactivity Model) created for accuracy and consistency with prior mechanistic work on ruthenium-based olefin metathesis.^66,67^ See the Methods and Section S2.1.3 for details.

First, whereas the original correlation-based design criterion ranked catalyst **17** ahead of **18**, in detailed mechanistic calculations described in the following, we should expect to see a significantly higher calculated barrier to metathesis for **17** than for **18**. A second objective of these calculations is to uncover why the correlation-based design criterion failed to rank the two catalysts correctly.

**Figure 3:**
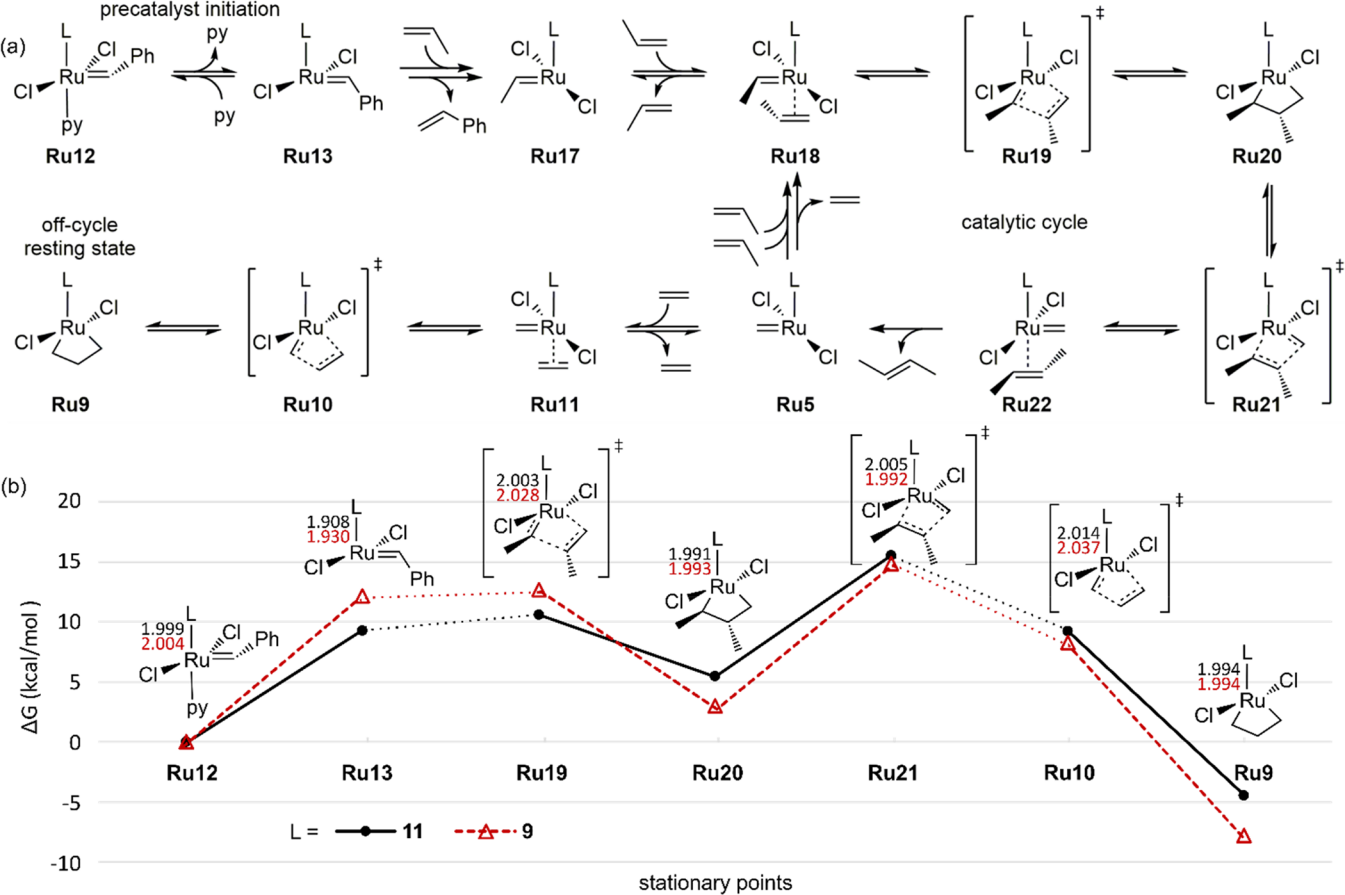
(a) Key intermediates and transition states of propene self-metathesis. (b) Gibbs free energies, relative to the pyridine precursor **Ru12**, calculated for key intermediates and transition states of propene self-metathesis in benzene (298.15 K) using catalysts **17** (L = **9**) and **18** (L = **11**). Only the formation of the most stable product (*E*-2-butene) was investigated. Stationary points not connected via a single elementary reaction step are linked by dotted lines. The Ru–L bond distance (Å) is given in black for **11** and in red for **9**.

To address the first objective, we calculated the free energies of the most relevant intermediates and transition states of 1-alkene metathesis mediated by **17** and **18** using propene as a computationally efficient model 1-alkene substrate (Figure 3). In addition, since the stability of the unsubstituted MCB (**Ru9**), via its correlation with *r*(Ru=CH_2_), is the underlying driving force in the de novo design, we also included **Ru9** and its corresponding transition state for cycloaddition/cycloreversion (**Ru10**). Although **Ru9** is an off-cycle intermediate, it is generated by the reaction of 14-electron methylidene intermediate **Ru5** with ethylene, which is a product of 1-alkene metathesis. **Ru9** is expected to be stable^80−83^ as well as easily accessible^81^ and may even constitute the resting state in 1-alkene metathesis catalyzed by phosphine-free catalysts.^67,79^ Indeed, **Ru9** is the most stable of the intermediates included in the present study (Figure 3). Thus, taking the difference between the free energy of **Ru9** and that of the transition state for cycloreversion **Ru21** as the relevant energetic span,^20^ we find that the free-energy barrier (Δ*G*^‡^) to metathesis is, as expected from the observed catalytic activities, higher (by 2.6 kcal/mol) for **17** (22.6 kcal/mol) than for **18** (20.0 kcal/mol).

An obvious contrast between the above energetic spans and the model behind the design criterion that ranked **17** ahead of **18** (Table 1) is that the former is calculated relative to **Ru9**, whereas the latter (Δ*E*^‡^ in Scheme 2) is predicted relative to 16-electron RuCl_2_(L)(L′)(=CH_2_) adducts (see below and the Methods). In fact, the prediction model behind the design criterion predicts (via the Ru=CH_2_ bond distance of **Ru5**) the stability of **Ru9** (as Δ*E*_MCB_) and assumes a correlation between Δ*E*_MCB_ and Δ*E*^‡^ (Scheme 2). In other words, not only does the prediction model not handle a change in the resting state, but the likely resting state is, in this case, an intermediate for which the energy is assumed (by the model) to covary perfectly with the energy of the rate-determining transition state.

The prediction model assumed 16-electron complexes that may be generated during catalysis to be the resting state, and its construction involved an average over such model complexes (L′ = PMe_3_, H_2_O, and CH_2_O; see the Methods).^31^ This assumption was based on prior calculations suggesting that 16-electron ruthenium–methylidene phosphine adducts are more stable than the unsubstituted MCB.^57^ Subsequent calculations have found such phosphine adducts to have stabilities comparable to or greater than those of the unsubstituted MCB intermediate.^84,85^ Therefore, to maintain correspondence with the prediction model, we first attempted to synthesize a phosphine-based precursor (starting from **14;**Scheme 3). Only when this synthesis failed did we turn to synthesizing phosphine-free precursors (starting from **1** and **16**; Scheme 3) that might have resting states different from that of 16-electron complexes^67,79^ and thus might be outside the applicability domain of the prediction model.

If, hypothetically, catalysts **17** and **18** had the resting state assumed by the design criterion, then our mechanistic calculations suggest that the ranking (**17** ahead of **18**) would have been qualitatively correct. In agreement with the correlations reported in Figure S1, the higher stability of **Ru9** for L = **9** compared to L = **11** versus the precursor (a 16-electron complex, albeit not a Ru–methylidene) transfers to a more stable transition state for cycloreversion of both the unsubstituted (**Ru10**) and the substituted (**Ru21**) MCB of productive propene self-metathesis using catalyst **17** (Figure 3). However, **17** only has a marginally lower barrier (by 0.7 kcal/mol) to cycloreversion (via **Ru21**) than **18**, suggesting a less-than-perfect correlation between the relative energies of **Ru21** and **Ru9** (for which **17** is more stable by 3.3 kcal/mol). In other words, although the relative order between **17** and **18** is maintained between MCB **Ru9** and the cycloreversion transition state **Ru21**, the latter is moderately destabilized vs **Ru9** for the new carbene **9**. This destabilization is not handled by the design criterion, and in the following we will briefly address the factors behind this destabilization.

The two most important and chemically meaningful properties known to correlate strongly with the design criterion (chemical productivity, taken as –*r*(Ru=CH_2_) as described in the Methods section) are the L-to-Ru σ-donation and the L-to-alkylidene steric repulsion.^31^ In the following, we will thus analyze the influence of these two properties, that is, to what extent **9**, as implied by the design criterion, is a better σ donor than **11** and has better activity-promoting steric repulsion toward the alkylidene. We start with an analysis of the electron donation from L-ligands **9** and **11**.

The frontier-orbital energies of **9** and **11** indicate that L-to-Ru σ-donation should be stronger for **9**: Whereas the energy of the lowest unoccupied molecular orbital (LUMO) is −1.16 eV for both ligands, that of the highest occupied molecular orbital (HOMO) is higher for **9** (−4.63 eV) than for **11** (−4.78 eV). Consistent with previous studies of σ-donor and π-acceptor properties of carbenes,^86−91^ this suggests that the two ligands have similar π-acceptor capacity but **9** is a better σ-donor than **11**, which is consistent with the design criterion.

**Figure 4:**
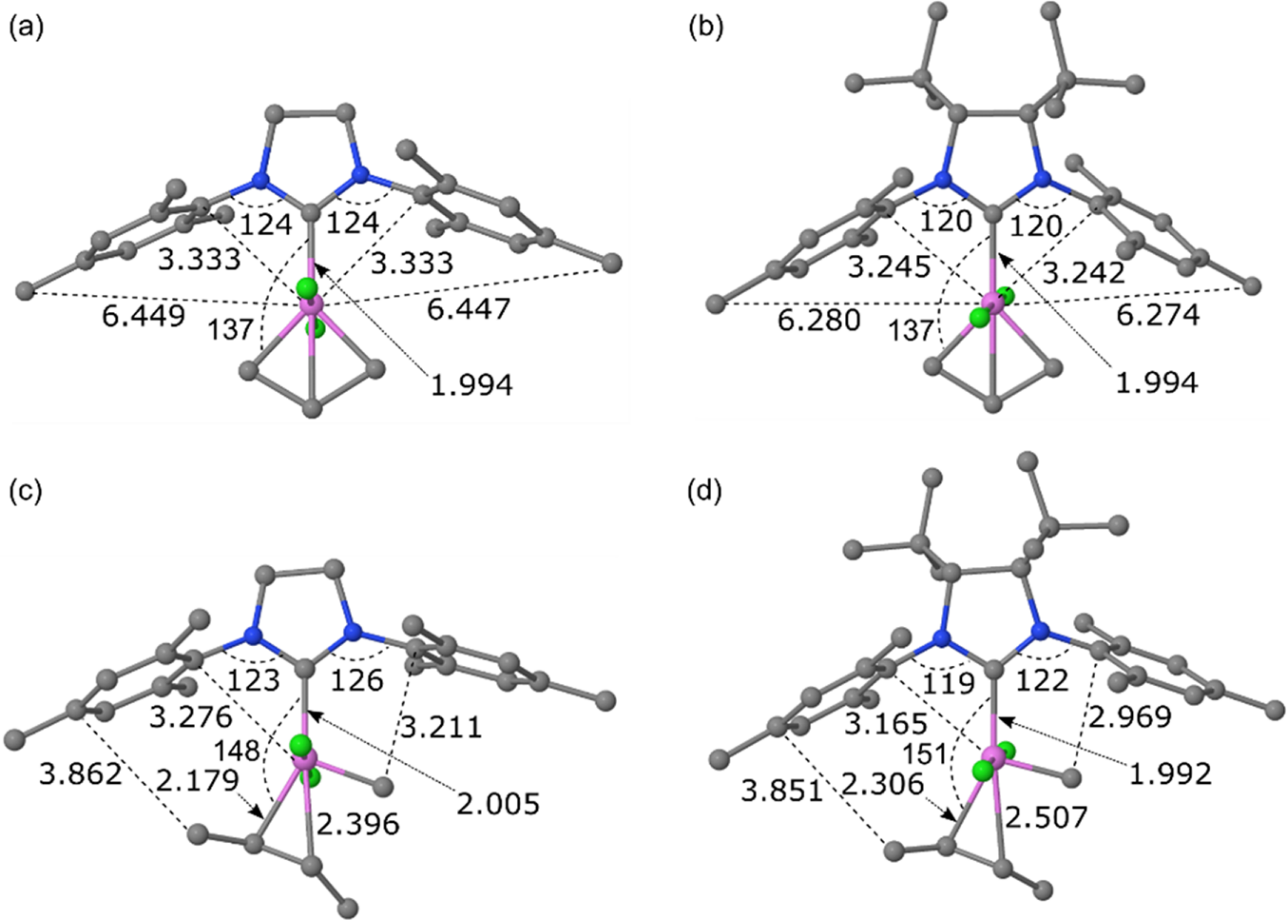
Ball-and-stick models of the optimized geometries of **Ru9** (L = **11** (a) and L = **9** (b)) and **Ru21** (L = **11** (c) and L = **9** (d)). See Figure 3 for Lewis structures. Also shown are selected bond distances (Å) and bond angles (deg). Hydrogen atoms have been omitted for clarity. Color coding: Ru: pink, Cl: green, C: gray, and N: blue.

Turning now to steric effects, the repulsion from the mesityl groups of carbene **9** is expected to be greater in complexes with ligands and substituents close to the carbene, and this repulsion should manifest itself in longer Ru–L distances (Figure 3). Whereas identical (**Ru9**) or almost identical (**Ru20**) Ru–L distances are obtained for the sterically least demanding intermediates (the MCBs), which are characterized by wide L–Ru-C angles (137° for **Ru9**; Figure 4), longer Ru–L bonds are typically found for the intermediates and transition states of carbene **9** with sharper L–Ru–C angles. This is the case, for instance, in the precursor **Ru12** and the corresponding 14-electron complex **Ru13** in which the Ru–benzylidene bond forms much sharper angles with the Ru–L bond (L–Ru–C = 98°–102°).

Due to the closer contact between the L-ligand and the alkylidene in active 14-electron complexes than between the L-ligand and the metallacycle in MCB intermediates, the steric bulk of the L-ligand is known to be a reactivity-driving property correlating with the design criterion.^31^ Sterically demanding L ligands are also known more generally to be beneficial for catalytic activity.^31,48,92,93^ Leading ruthenium-based catalysts are indeed based on sterically demanding N-heterocyclic carbenes, such as **11**,^47^ or cyclic alkyl amino carbenes.^67,95^ It is thus unsurprising that the current de novo design led to carbenes that are bulkier than **11**. However, the question is whether the steric-related relationship encoded in the design criterion still holds beyond **11**. The calculated bond dissociation free energies (BDFEs) of the two carbene ligands **9** (35.5 kcal/mol) and **11** (38.8 kcal/mol) in their respective catalyst precursors **17** and **18** indicate that **9** might be outside the applicability domain of the prediction model. Despite its better σ-donating capacity, carbene **9** is bulky enough to weaken the Ru–L bond by almost 10% and to destabilize **Ru21** versus **Ru9** on going from carbene **11** to the bulkier **9** by 2.6 kcal/mol. This is consistent with the above-described more difficult installation on ruthenium observed for this carbene and, in particular, with the fact that syntheses starting from the more sterically demanding complexes **1** and **14** (Scheme 3) did not lead to isolable products.

One might have expected the destabilization of **Ru21** versus **Ru9** on going to the bulkier carbene **9** to be due in part to the added steric repulsion of the propene versus the ethylene substrate. In other words, for the larger carbene **9**, the propene self-metathesis cycloreversion transition state **Ru21** might be destabilized by specific steric interactions between the forming *E*-2-butene and the relatively narrow catalytic pocket formed by the two N-bound mesityl groups of **9** (Figure 3). Perhaps surprisingly, this does not seem to be the case: Although closer butene–mesityl contacts are seen for catalyst **17** than for **18** in **Ru21**, the former is slightly more stable relative to the precursor as is the corresponding transition state **Ru10** for cycloaddition/cycloreversion of the unsubstituted MCB. In other words, there seems to be little destabilizing impact of the larger steric bulk of propene versus ethylene on the cycloreversion transition state.

In summary, detailed mechanistic calculations suggest that the de novo designed carbene **9** does indeed score higher on the two main reactivity-driving factors, namely, the L-to-Ru σ-donation and the L-to-alkylidene steric repulsion, built into the prediction model of the design criterion. However, the weaker Ru–L bond calculated for **9** indicates that the steric bulk of this carbene in addition to the catalysis-enhancing effects captured by the prediction model also has undesired consequences not handled by the model. Foremost among these consequences is that a phosphine-stabilized catalyst precursor could not be made and the pyridine-stabilized precursor **17** that eventually was obtained is less stable than the reference compound **18**. In fact, DFT calculations show that **17** is significantly less stable than the unsubstituted MCB **Ru9** (Figure 3) and therefore adopts a catalyst resting state different from that of the 16-electron ruthenium–phosphine adducts assumed by the prediction model. A fitness function augmented by a measure of the stability of the phosphine-based precursor bearing the candidate ligand could have prevented the automated de novo procedure from ranking bulky ligands such as **9** that highly. More generally, a more sophisticated prediction model including correlations between the selected intermediate (here, **Ru9**) and all other stationary points (including the precursor) along the catalytic pathway, such as in LFESR,^24−26^ would presumably also have detected and reacted appropriately to the switch in the resting state caused by a destabilized precursor.

## Conclusions

The usefulness of de novo molecular design is essentially decided by the accuracy and computational cost of the fitness function. The hypothetical perfect computational fitness function for the design of molecular catalysts would be based on the accurate knowledge of the pathways of both the desired and the competing undesired reactions. This ideal approach is not feasible in de novo design for the foreseeable future. Indeed, de novo design studies of homogeneous catalysts have so far relied heavily on correlation-based fitness functions,^3,58^ albeit without synthesis and testing of the thus designed catalysts. In the present study, a previously reported correlation-based design criterion (a QSAR-type prediction model) was used in de novo design of NHC ligands for ruthenium-based olefin metathesis catalysts, with an experimental follow-up of the most promising designed candidate carbene (**9**). Although a catalyst precursor (**17**) bearing ligand **9** eventually could be synthesized, this precursor was pyridine-stabilized instead of the phosphine stabilization assumed by the prediction model. Although **17** proved to be active for metathesis, its catalytic performance was inferior to that of the known pyridine-stabilized third-generation Grubbs-type reference catalyst **18**.

Detailed computational analysis suggested that the de novo designed carbene ligand **9** does indeed offer more L-to-Ru σ-donation and L-to-alkylidene steric repulsion, which are the two main reactivity-driving factors built into the original correlation-based design criterion. The analysis also indicated, perhaps counterintuitively, that the use of ethylene as a model substrate in the prediction model does not introduce significant errors. Thus, under the main assumption behind the current design criterion that a stable MCB leads to a low barrier to metathesis, the analysis largely supported the simple distance-based fitness (−*r*(Ru=CH_2_)) used to predict compounds with stable MCBs.

Clues to understanding the disappointing catalytic performance of **17** are to be found instead in the lower calculated L–Ru BDFE of carbene **9** than the carbene (**11**) of the reference catalyst **18** and the related failure to obtain a phosphine-based precursor. Whereas the prediction model of the de novo design relied on 16-electron RuCl_2_(L)(L′)(=CH_2_) adducts as the resting state, a very different intermediate, that is, the unsubstituted MCB, turned out to be the likely resting state for **17**. Thus, this catalyst does not represent the ideal but rather the seemingly only possible experimental validation of the de novo prediction of **9** as a ligand candidate. However, that this compromise (pyridine instead of phosphine) had to be made suggests that the de novo design criterion or fitness function failed to account for negative consequences of the steric bulk of **9**. Already a relatively simple fitness function augmented by a measure of the stability of the phosphine-based precursor bearing the candidate ligand could presumably have prevented **9** from being predicted as a promising candidate ligand.

Thus, the main lesson to be drawn from this study is that design criteria for de novo catalyst design should incorporate information from more than a single intermediate or a single relative energy. If correlations with the rest of the catalytically relevant intermediates and transition states are established prior to the design itself, such as in LFESR,^24−26^ a single calculation may still suffice to obtain the fitness of a candidate. The possibilities and limitations of this approach should be tested, preferably against experimental validation, in future de novo catalyst design studies.

## Data Availability

Parameters, data, and software used in the de novo design is available at 10.5281/zenodo.7762776. A data set collecting the results from the Mechanistic DFT Investigation section is available from the ioChem-BD repository;^96^ it can be accessed via 10.19061/iochem-bd-6-292. Commercial molecular modeling software used to produce such data, i.e., Gaussian^97^ and Spartan,^98^ can be obtained from the corresponding vendors.

## Supporting Information Available

Experimental details, NMR spectra, computational details and supplementary computational results and data (PDF)
Computed molecular Cartesian coordinates (XYZ)
Compounds (ZIP)

## Supplementary Material

ci3c01649_si_001.pdf

ci3c01649_si_002.xyz
