# Supplementary material for: Automated de Novo Design of Olefin Metathesis Catalysts: Computational and Experimental Analysis of a Simple Thermodynamic Design Criterion
Source: J Chem Inf Model. 2024 Jan 10;64(2):412–24. doi: 10.1021/acs.jcim.3c01649 (PMC10806812; doi:10.1021/acs.jcim.3c01649)
Supplement: Supplementary file 1 — ci3c01649_si_001.pdf [file ci3c01649_si_001.pdf]

Supporting Information

for

Automated De Novo Design of Olefin Metathesis Catalysts: Computational and Experimental Analysis of a Simple Thermodynamic Design Criterion

Marco Foscato,* Giovanni Occhipinti,* Sondre H. Hopen Eliasson, Vidar R. Jensen*

Department of Chemistry, University of Bergen, Norway

TABLE OF CONTENTS

S1 Previously Published Correlation S2
S1.1 Stability of MCB Intermediate **Ru9** vs Cycloreversion Transition State **Ru10** S2
S1.2 Length of Ru=CH_2 at **Ru5** vs Average Stability of MCB Intermediate **Ru9** S3
S1.3 Sensitivity of Ru=C Bond Length to Computational Model S5
S2 Computational Part S9
S2.1 Computational Details S9
S2.1.1 Prediction Model 1 ..... S9
S2.1.2 Prediction Model 2 ..... S9
S2.1.3 Reactivity Model S10
S2.2 Computational Data S12
S2.2.1 De Novo Design Experiments S12
S2.2.2 Productivities Estimated with Prediction Model 2. ..... S12
S2.2.3 Reaction Energy Profiles for Precatalyst **17** and **18** S14
S3 Experimental Part S16
S3.1 Experimental Details S16
S3.2 Preparation of Ruthenium Complex **17** S16
S3.3 Self-Metathesis of Neat 1-Octene with 100 ppm of Catalyst at Room Temperature (Figure 2, Main Paper) S16
S3.4 Self-Metathesis of Neat 1-Octene with 1 ppm of Catalyst at 60 °C S16
S3.5 NMR Spectra of **17** S17
S4 References S20

S1 Previously Published Correlation

S1.1 Stability of MCB Intermediate Ru9 vs Cycloreversion Transition State Ru10

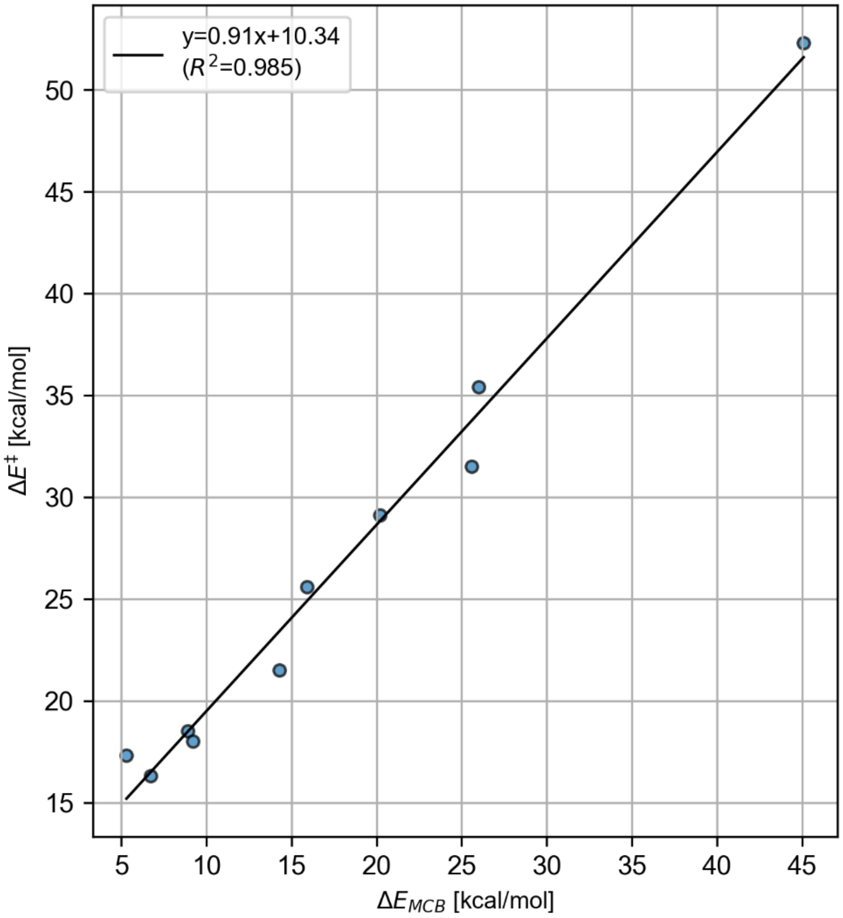

Figure S1. Correlation between the relative energy of metallacyclobutane **Ru9** ($\boldsymbol{\Delta E_{MCB}}$) and that of the cycloreversion transition state **Ru10** ($\boldsymbol{\Delta E^{\ddagger}}$). Values taken from Ref. 1 and reported in Table S1.

Table S1. Relative Energies (kcal/mol Relative to Ru8) Taken from Table 1 of Ref. 1.[a]

L	PCy_3	PMe_3	PH_3	PH_3	PMe_3	H_2IMes[a]	H_2IH[a]	H_2IH[a]	PCy_3	PH_3
L'	PCy_3	PMe_3	PH_3	PMe_3	PH_3	PCy_3	PMe_3	PH_3	H_2IMes[a]	H_2IH[a]
Ru8	0	0	0	0	0	0	0	0	0	0
**Ru9** (ΔE_{MCB})	8.9	20.2	14.3	25.6	9.2	5.3	15.9	6.7	26	45.1
**Ru10** ($\Delta E^{\ddagger}$)	18.5	29.1	21.5	31.5	18	17.3	25.6	16.3	35.4	52.3

[a]Catalyst identifiers are defined in the original publication. See Ref. 1 for details.

S1.2 Length of Ru=CH_2 at Ru5 vs Average Stability of MCB Intermediate Ru9

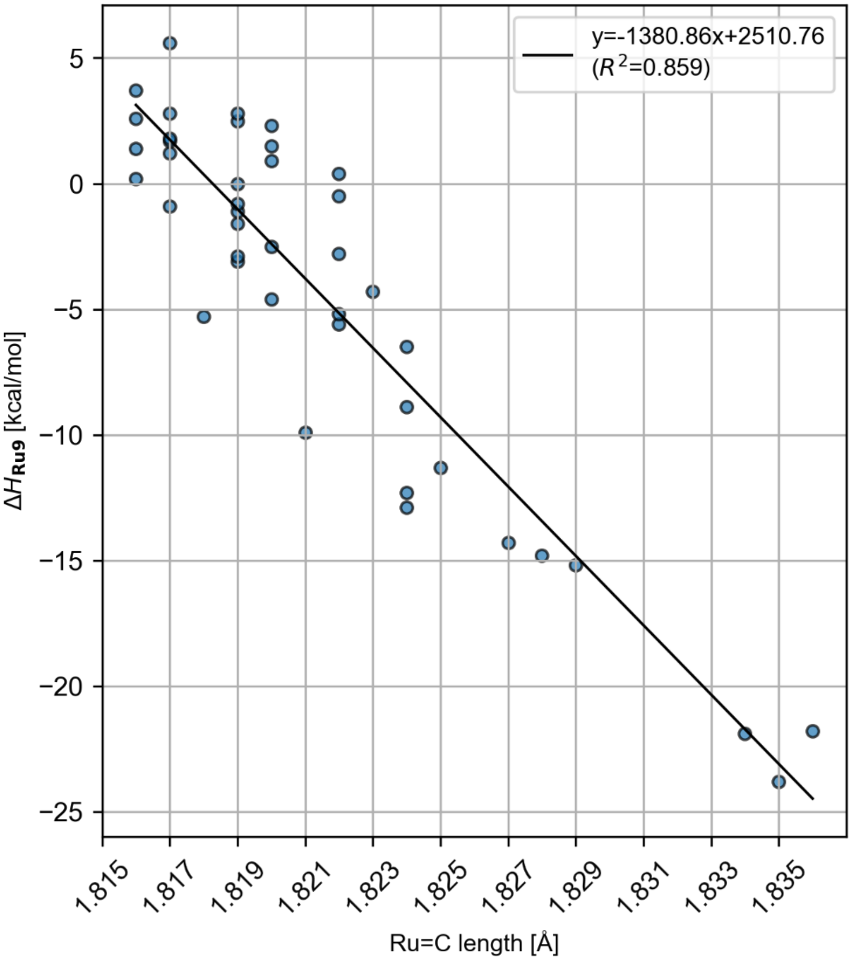

Figure S2. Correlation plot between the enthalpic components of the stability of metallacyclobutane (MBC) **Ru9** and the length of the Ru=CH_2 bond at **Ru5**. Values taken from Ref. 2 and reported in Table S2.

Table S2. Ru=CH_2 Bond Lengths at Ru5 and Taken from the Supporting Information of Ref. 2. Refer to the Original Publication for Further Details.

Catalyst ID[a]	Ru=C [Å]	Calculated productivity ΔH_{Ru9} [kcal/mol]
CO	1.835	-23.8
P16	1.834	-21.9
CSe	1.836	-21.8
NC1	1.829	-15.2
P13	1.828	-14.8
S5	1.827	-14.3
P1	1.824	-12.9
As1	1.824	-12.3
P8	1.825	-11.3
S3	1.821	-9.9
As2	1.824	-8.9
A1	1.824	-6.5

S2	1.822	-5.6
N1	1.818	-5.3
P4	1.822	-5.2
N2	1.82	-4.6
A28	1.823	-4.3
O4	1.819	-3.1
P7	1.819	-2.9
A2	1.822	-2.8
H3	1.82	-2.5
A29	1.819	-1.6
A37	1.819	-1.1
A27	1.817	-0.9
A11	1.819	-0.8
A35	1.822	-0.5
N4	1.819	0
A23	1.816	0.2
A26	1.822	0.4
A6	1.82	0.9
A14	1.817	1.2
A25	1.816	1.4
A3	1.82	1.5
A15	1.817	1.7
A13	1.817	1.8
A10	1.82	2.3
A24	1.819	2.5
A12	1.816	2.6
A5	1.819	2.8
A16	1.817	2.8
A20	1.816	3.7
A8	1.817	5.6

[a]Catalyst identifiers are defined in the original publication. See Ref. 2 for details.

S1.3 Sensitivity of Ru=C Bond Length to Computational Model

To assess the sensitivity of the Ru=C bond length descriptor to basis set and solvation model, the geometry of **Ru5** for a representative selection of ligands among those in the training set of Ref. 2 (and listed in Table S2) was re-optimized with three variations of the computational protocol here defined as *Prediction Model 1* (see Computational Details section). (i) an extended basis set, def2TZVPP, instead of LANL2DZ, (ii) implicit solvation in benzene as from the SMD model,[3] and (iii) the combination of def2TZVPP basis set with SMD solvation in benzene.

Although the values are numerically different (Table S3) the trend is essentially retained. The series obtained from each variation of *Prediction Model 1*, in fact, show high correlation coefficients with the series obtained from the original *Prediction Model 1* (see Figure S3, Figure S4, and Figure S5). Consequently, the correlation between the Ru=C bond length and the computed productivity remains high (R^2>0.92) for each alternative computational model (Figure S6, Figure S7, and Figure S8). Importantly, the Ru=C bond distance obtained for the de novo designed ligand **9** is consistently the shortest.

Overall, these results suggest that *Prediction Model 1* is relatively insensitive to extending the basis set or to inclusion of solvent effects. The higher computational cost that would derive from the application of such refinements are not justified.

Table S3. Sensitivity to Basis Set and Solvation Model of Ru=CH_2 Bond Lengths of Ru5.

Catalyst ID	Ru=C [Å]			
	Prediction Model 1	*Prediction Model 1* with def2TZVPP	*Prediction Model 1* with SMD(benzene)	*Prediction Model 1* with def2TZVPP and SMD(benzene)
A12[a]	1.816	1.788	1.816	1.785
A13[a]	1.817	1.788	1.815	1.785
A15[a]	1.817	1.789	1.816	1.786
A37[a]	1.819	1.791	1.819	1.788
As2[a]	1.824	1.795	1.821	1.792
CO[a]	1.835	1.808	1.833	1.804
H3[a]	1.820	1.789	1.818	1.784
NC1[a]	1.829	1.802	1.827	1.796
P16[a]	1.834	1.808	1.831	1.802
P4[a]	1.822	1.797	1.821	1.794
P8[a]	1.825	1.800	1.824	1.796
9[b]	1.813	1.784	1.812	1.781

[a]Catalyst identifiers are defined in the original publication. See Ref. 2 for details. [b]Catalyst designed in this work.

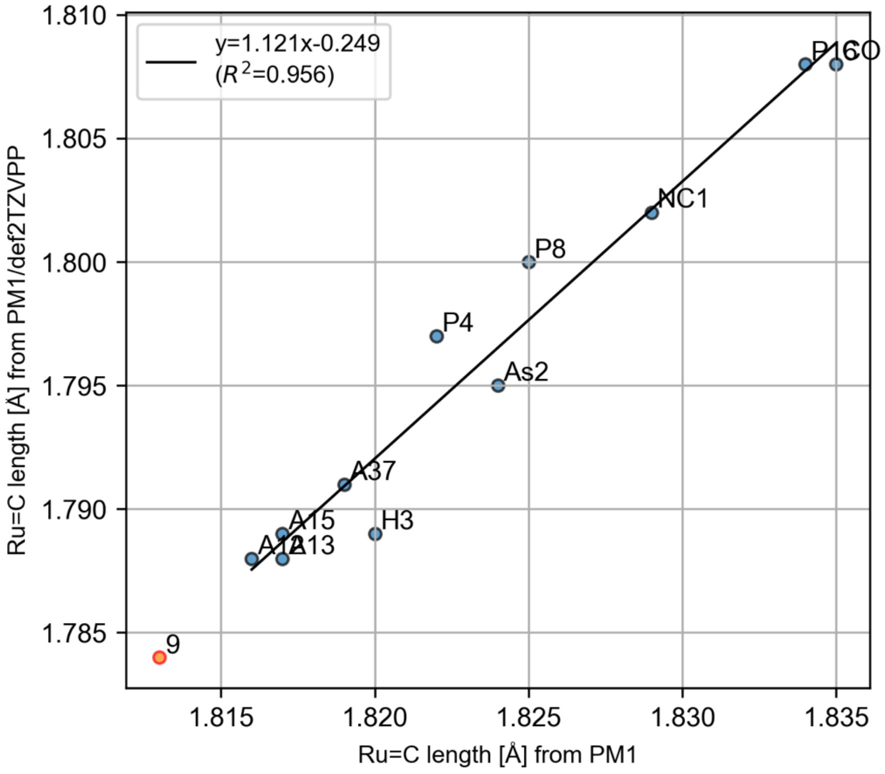

Figure S3. Correlation plot for Ru=C bond lengths in molecular models of **Ru5** optimized with *Prediction Model 1* (*PM1* for brevity) and its variation using def2TZVPP basis set. Data from Table S3. The red point represents the ligand designed in this work, i.e., ligand **9**.

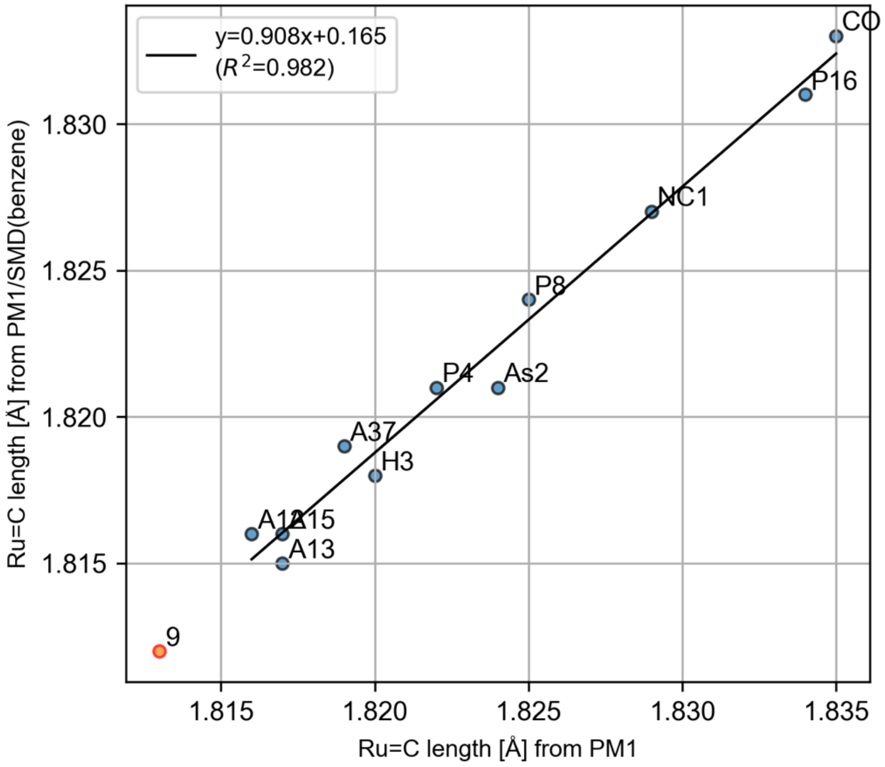

Figure S4. Correlation plot for Ru=C bond lengths in molecular models of **Ru5** optimized with *Prediction Model 1* (*PM1* for brevity) and its variation using SMD(benzene) solvation model. Data from Table S3. The red point represents the ligand designed in this work, i.e., ligand **9**.

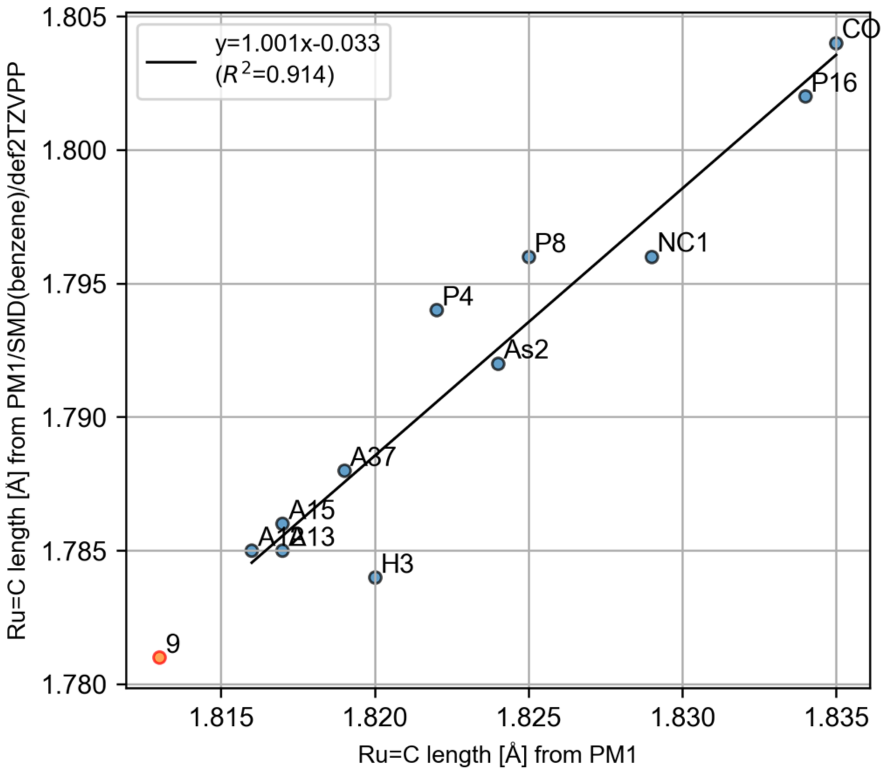

Figure S5. Correlation plot for Ru=C bond lengths in molecular models of **Ru5** optimized with *Prediction Model 1* (*PM1* for brevity) and its variation using def2TZVPP basis set and SMD(benzene) solvation model. Data from Table S3. The red point represents the ligand designed in this work, i.e., ligand **9**.

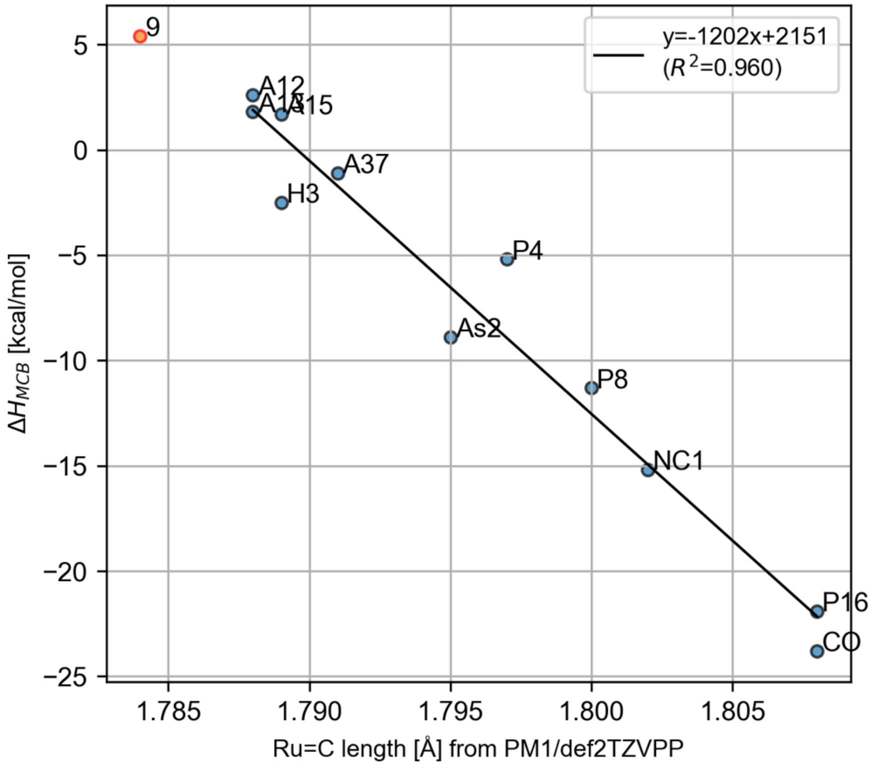

Figure S6. Correlation between the enthalpic components of the stability of metallacyclobutane (MBC) **Ru9** (values taken from Ref. 2 and reported in Table S2) and the length of the Ru=CH_2 bond at **Ru5** when optimized with the variation of *Prediction Model 1* (*PM1* for brevity) using def2TZVPP basis set (data from Table S3).

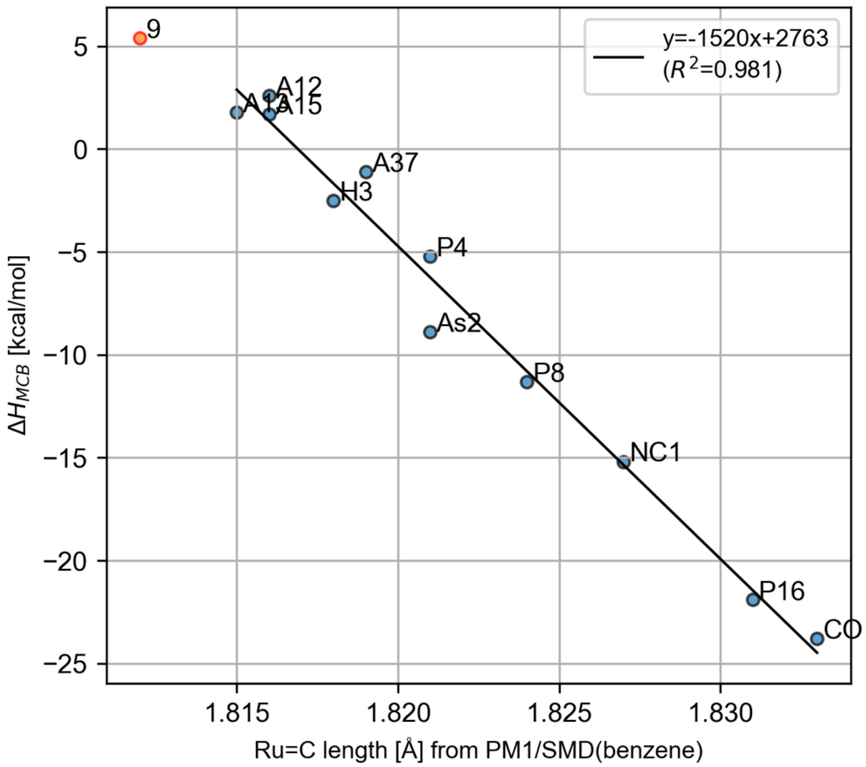

Figure S7. Correlation between the enthalpic components of the stability of metallacyclobutane (MBC) **Ru9** (values taken from Ref. 2 and reported in Table S2) and the length of the Ru=CH_2 bond at **Ru5** when optimized with the variation of *Prediction Model 1* (*PM1* for brevity) using SMD(benzene) solvation model (data from Table S3).

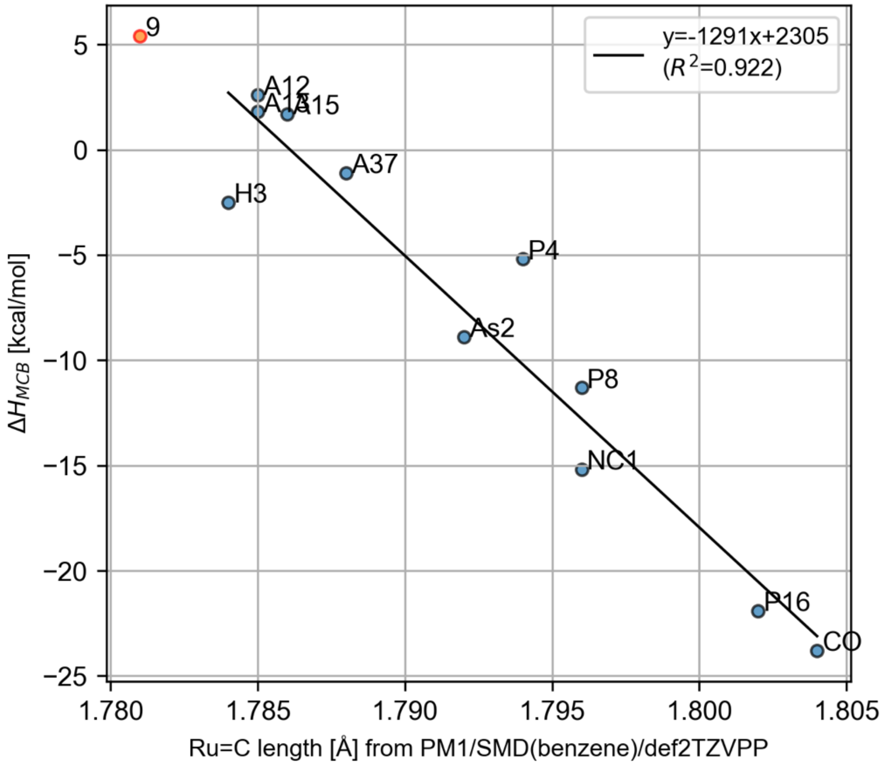

Figure S8. Correlation between the enthalpic components of the stability of metallacyclobutane (MBC) **Ru9** (values taken from Ref. 2 and reported in Table S2) and the length of the Ru=CH_2 bond at **Ru5** when optimized with the variation of *Prediction Model 1* (*PM1* for brevity) using def2TZVPP basis set and SMD(benzene) solvation model (data from Table S3).

S2 Computational Part

S2.1 Computational Details

S2.1.1 Prediction Model 1

The prediction model used in de novo design experiments is based on the findings of ref. 2 (i.e., correlation between Ru=CH_2 distance and productivity) and was designed specifically for automated catalyst design using DFT-quality molecular models while using the least possible computational resources. The initial molecular model of each candidate visited during evolutionary experiments was constructed by assembling the 3D structure of its building blocks, i.e., the molecular fragments that define the structure in the genetic algorithm.[4] The molecular geometry was then relaxed by performing a conformational search with the potential energy surface smoothing and search method available in Tinker (v6.3).[5] The potential energy used in this calculation included only the van der Waals term of the universal force field (UFF),[6] and operated in the torsional space defined by all rotatable bonds connecting two building blocks. The resulting geometry was analyzed to exclude the presence of atom clashes and submitted to DFT-driven geometry optimization. These DFT calculations were performed with Gaussian 09-B.01[7] or 16-C.01,[8] using one version consistently within a single experiment. For consistency with previous work on which the prediction model is based upon,[2] molecular geometries were optimized using the "pure" DFT functional OLYP (a combination of Handy's OPTX[9,10] exchange functional and the correlation functional by Lee, Yang, and Parr (LYP)).[11,12] All atoms were described by LANL2DZ basis sets, which consists of ECP for core electrons plus associated double-ξ basis set for the valence electrons.[13–15] Integration used the Gaussian's *ultrafine* grid, and self-consistent field procedure was converged to Gaussian's default criteria (RMS change in density matrix < $1.0{\cdot}10^{-8}$, max. change in density matrix = $1.0{\cdot}10^{-6}$). The geometry optimization did not exploit symmetry and was continued until satisfaction of the *tight* convergence criteria (max. force $1.5{\cdot}10^{-5}$, RMS force $1.0{\cdot}10^{-5}$, max. displacement $6.0{\cdot}10^{-5}$, RMS displacement $4.0{\cdot}10^{-5}$). A complete definition of the DFT protocol leading to the final geometry optimization is available at https://doi.org/10.5281/zenodo.7762776, where the complete machinery for generating input files is also available as open source.

From the DFT-optimized geometry the Ru=C bond length was calculated and, its negated value, collected to be used as fitness value in the genetic algorithm. Geometries were also checked for correctness. In particular, we ensured compatibility (i.e., presence of bonds, but not bond types) between the connectivity of the input to DFT and the one obtained from OpenBabel's[16] connectivity perception from the Gaussian output file. Moreover, to ensure consistency of the DFT-modelled geometry with the expected *trans*-Cl geometry of **Ru5**, candidates were accepted only when respecting the following geometrical requirements: i) interatomic distance to Ru greater than 2.7 Å for all atoms external to the fragment C_{NHC}–Ru(=CH_2)(Cl)Cl, ii) average Ru–Cl bond distance shorter than 2.5 Å, iii) average Cl–Ru–C_{NHC} angle greater than 90°, iv) minimum dihedral angle H–C=Ru–C_{NHC} lower than 20°.

With the exclusion of third-parties software, all parameters and software tools are available at https://doi.org/10.5281/zenodo.7762776.

S2.1.2 Prediction Model 2

According to the previous work from ref. 2, the catalyst productivity was estimated by the stability of the MCB intermediate **Ru9** relative to the averaged energy of three models for the 16-electron species **Ru8**. The latter was calculated as the average of **Ru8** models where L' = PMe_3, H_2O, or CH_2O:

$$Productivity = \overline{\Delta H}_{\mathbf{Ru8}} - \Delta H_{\mathbf{Ru9}} \qquad \text{Equation 1}$$

$$\overline{\Delta H}_{\mathbf{Ru8}} = \frac{\Delta H_{\mathbf{Ru8},PMe_3} + \Delta H_{\mathbf{Ru8},H_2O} + \Delta H_{\mathbf{Ru8},CH_2O}}{3} \qquad \text{Equation 2}$$

Enthalpy differences are given with respect to the 14-electron active complexes **Ru5**, which cancels out in Equation 1, thus

$$\Delta H_{\mathbf{Ru9}} = H_{\mathbf{Ru9}} - (H_{\mathbf{Ru5}} + H_{ethene}) \qquad \text{Equation 3}$$

and

$$\Delta H_{\mathbf{Ru8},L'} = H_{\mathbf{Ru8},L'} - (H_{\mathbf{Ru5}} + H_{L'}) \qquad \text{Equation 4}$$

where the enthalpy of a given molecule, $H_{\boldsymbol{X}}$, is calculated as the sum of the electronic energy, $E_{B3LYP,\boldsymbol{X}}$, and the enthalpy correction, $H^{corr}_{OLYP,\boldsymbol{X}}$:

$$H_{\boldsymbol{X}} = E_{B3LYP,\boldsymbol{X}} + H^{corr}_{OLYP,\boldsymbol{X}} \qquad \text{Equation 5}$$

The molecular models of relevant MCB complexes **Ru9** and the 16-electron species **Ru8** were constructed in Spartan'08[17] and conformational searches were performed using semiempirical method PM3.[18] Low-energy conformers were then taken as input structures for geometry optimization using the same DFT protocol described above for *Prediction Model 1*, except for Gaussian09's default *fine* integration grid and default convergence criterion (max. force $4.5{\cdot}10^{-4}$, RMS force $3.0{\cdot}10^{-4}$, max. displacement $1.8{\cdot}10^{-3}$, RMS displacement $1.2{\cdot}10^{-3}$) in accordance with the original work.[2] The nature of the final stationary points was confirmed by the analysis of the eigenvalues of the analytically calculated Hessian matrix. The ideal gas, rigid-rotor

and harmonic oscillator approximations were used to compute the thermochemical corrections required to get the enthalpy at 298.15 K used in the productivity calculation ($H_{OLYP,\boldsymbol{X}}^{corr}$ in Equation 5).

Single-point energies ($E_{B3LYP,\boldsymbol{X}}$ in Equation 5) were obtained using the hybrid functional B3LYP[19] and an upgraded basis set from the geometry optimizaton. For ruthenium, the Hay and Wadt primitive basis set (5s,6p,4d)[20] was contracted to [4s,4p,3d]. For the other (non-Ru) elements, a diffuse s-function was added throughout, whereas, in addition, a diffuse p-function was added to all non-hydrogen elements. The diffuse s-functions of the non-hydrogen elements were added in an even-tempered manner, whereas the s-exponent for hydrogen and the p-exponents were modified.[21] Polarization functions were added for all atoms; a p-function for hydrogen and a d-function for the other elements. The self-consistent field convergence criterion was set to *SCF(Conver=5)* (RMS change in density matrix < $1.0{\cdot}10^{-5}$, max. change in density matrix = $1.0{\cdot}10^{-3}$) and otherwise default Gaussian 09 settings were used.

Energies ($E_{B3LYP,\boldsymbol{X}}$) and thermal correction to the enthalpy ($H_{OLYP,\boldsymbol{X}}^{corr}$) for all molecular models involved in the calculation of the productivities are reported in Table S4. The input files and the results of the geometry optimizations and single point calculations are available in the ioChem-BD repository[22,23] at https://doi.org/10.19061/iochem-bd-6-292.

S2.1.3 Reactivity Model

Model Building. The construction of molecular structures, conformational searches, and preliminary strain relaxations of molecular models were performed with Spartan18[24] using its implementation of Merck's force field (MMFF94)[25] and of the semi-empirical method PM6.[26] These calculations were carried out in conjunction with manually-set geometrical constraints in the first coordination sphere of ruthenium, to preserve geometrical features inaccurately described by empirical and semiempirical methods. All density functional theory (DFT) calculations were performed with the Gaussian 16 suite of programs, version 16 C.01.[27]

Geometry Optimization. Geometry optimization was performed using the Gaussian 16 implementation of the generalized-gradient approximation (GGA) functional of Perdew, Burke and Ernzerhof (PBE),[28,29] including Grimme's D3 empirical dispersion term[75] in conjunction with revised Becke-Johnson damping (overall labelled PBE-D3M(BJ) for brevity).[76]

All atoms except ruthenium were described by Dunning's correlation-consistent valence double-ζ plus polarization basis sets (cc-pVDZ),[30,31] as retrieved from the EMSL basis set exchange database.[32,33] Ruthenium was described with the Stuttgart 28-electron relativistic effective core potential (ECP28MDF retrieved from the Stuttgart/Cologne group website)[34] in combination with the correlation-consistent valence double-ζ plus polarization basis set (cc-pVDZ-PP)[34] retrieved from EMSL basis set exchange database.[32,33]

The Gaussian 16 *ultrafine* grid was explicitly specified for numerical integration (keyword int=ultrafine), which implies that this grid was used also for the analytical Hessian calculations. Geometries were optimized using tight convergence criteria (max. force $1.5{\cdot}10^{-5}$ a.u., RMS force $1.0{\cdot}10^{-5}$ a.u., max. displacement $6.0{\cdot}10^{-5}$ a.u., RMS displacement $4.0{\cdot}10^{-5}$ a.u.), without symmetry constraints, using the following convergence criteria for the self-consistent field (SCF) optimization procedure: RMS change in density matrix < $1.0{\cdot}10^{-9}$, max. change in density matrix < $1.0{\cdot}10^{-7}$.

All stationary points were characterized by the eigenvalues of the analytically calculated Hessian matrix, confirming the absence (for minima) or presence of a single negative eigenvalue (for transition states). The translational, rotational, and vibrational components of the thermal corrections to enthalpies and Gibbs free energies were calculated within the ideal-gas, rigid-rotor, and harmonic oscillator approximations considering a temperature of 298 K, except that all frequencies below 100 cm^{-1} were shifted to 100 cm^{-1} when calculating the vibrational component of the entropy (i.e., the quasi-harmonic oscillator approximation)[35] to prevent the asymptotic behavior of the harmonic approximation with modes of very low frequencies.

Energies (E_{PBE}) and thermal correction ($G_{\mathrm{PBE}\ qh}^{298.15\mathrm{K}}$) for all molecular models are reported in Table S5.The input files and the results of the geometry optimizations and Hessian calculations are available in the ioChem-BD repository[22,23] at https://doi.org/10.19061/iochem-bd-6-292.

Single-Point Energy Calculations. All single-point energy calculations were performed with the Gaussian 16 implementation of the generalized gradient approximation (GGA) functional of Perdew, Burke and Ernzerhof (PBE)[28,29] including Grimme's D3 empirical dispersion term[36] with revised Becke-Johnson damping (overall labelled PBE-D3M(BJ) for brevity).[37]

Ruthenium was described by the ECP28MDF relativistic effective core potential[34] accompanied by a correlation-consistent valence quadruple-ζ plus polarization basis set (ECP28MDF_VQZ),[34] both obtained from the Stuttgart/Cologne Group website.[38] Carbon and hydrogen atoms were described by valence quadruple-ζ plus polarization (EMSL: cc-pVQZ)[32,33] basis sets.[31] Nitrogen and chlorine atoms were described by the valence quadruple-ζ plus polarization augmented with diffuse functions (EMSL: aug-cc-pVQZ),[30,32,33,39] Electrostatic and non-electrostatic solvation effects in benzene were taken into account by using the polarizable continuum model (PCM) in combination with the "Dis", "Rep", and "Cav" keywords and the built-in program values (dielectric constant, number density, etc.).[40–43] The solute cavity was constructed using the united atom topological model with atomic radii optimized for Hartree−Fock (termed "UAHF").[43–46] Numerical integrations were performed with the *ultrafine* grid of Gaussian 16, and the self-consistent field (SCF) density-based convergence criterion was set to 10^{-5} (RMS change in density matrix < $1.0{\cdot}10^{-5}$, max. change in density matrix = $1.0{\cdot}10^{-3}$).

Energy values ($E^{C_6H_6}_{\text{PBE-D3M(BJ)}}$) for all molecular models are reported in Table S5.The input files and the results of the single point energy calculations are available in the ioChem-BD repository[22,23] at https://doi.org/10.19061/iochem-bd-6-292.

Calculation of Gibbs Free Energies. Gibbs free energies were calculated at 298.15 K according to Equation 6:

$$G^{C_6H_6\ 298K\ [1M]}_{\text{PBE-D3M(BJ)}} = E^{C_6H_6}_{\text{PBE-D3M(BJ)}} + G^{C_6H_6\ 298.15K}_{\text{PBE}\ qh} + G^{\ 298.15K}_{1atm\rightarrow 1M} \qquad \text{Equation 6}$$

where $E^{C_6H_6}_{\text{PBE-D3M(BJ)}}$ is the potential energy resulting from single-point calculation with PBE-D3M(BJ), and include the contributions from the implicit solvation model; $G^{C_6H_6\ 298K}_{\text{PBE}\ qh}$ is the thermal correction to the Gibbs free energy calculated at the geometry optimization level with the quasi-harmonic approximation at 298.15 K; and $G^{\ 298.15K}_{1atm\rightarrow 1M}$ is the standard state correction corresponding to 1 M solution (but exhibiting infinite-dilution, ideal-gas-like behavior), which is equal to 1.89 kcal mol^{-1} (= RT·ln(24.46)) at room temperature. Table S5 reports Gibbs free energy values ($G^{C_6H_6\ 298.15K\ [1M]}_{\text{PBE-D3M(BJ)}}$) and the relative values ($\Delta G^{C_6HCl_6\ 298.15K\ [1M]}_{\text{PBE-D3M(BJ)}}$) calculated with respect to the pyridine-stabilized precatalyst **Ru12**. **Ru12_9** and **Ru12_11** are, thus, the reference points for the species bearing the ligands **9** and **11** respectively.

Natural Bond Orbital (NBO) Analyses. The natural bond orbital analyses were performed with the NBO7 software,[47] using the electron density of the single-point energy calculations as input.

The overall electron donation of the carbene ligands in the ruthenium species **Ru9**, **Ru19**, and **Ru21** was calculated as the natural charge of the fragment $Cl_2Ru(CH_2)_3$ for **Ru9**, and that of the fragment $Cl_2Ru(CH(CH_3)CH(CH_3)CH_2)$ for **Ru19**, and **Ru21**.

The carbene π-back-donation in the ruthenium complex **Ru9** has been estimated as the difference between the occupancy on the lone vacancy (LV) at the carbene atom in the complex **Ru9** and that in the isolated ligand fragment having the same geometry as in complex **Ru9**. The carbene σ-donation has been calculated as the sum of the overall donation from natural charge analysis (see above) and the π-backdonation.

To ensure a comparable set of orbitals between the complexes and the 'frozen' ligand fragments, the Lewis structures were explicitly required (via the $CHOOSE input section) to have a lone pair (LP) at the carbene carbon atom as well as on the adjacent N-atoms.

S2.2 Computational Data

S2.2.1 De Novo Design Experiments

The input data, parameters, software tools, and output data pertaining the evolutionary de novo design experiments are available at https://doi.org/10.5281/zenodo.7762776.

S2.2.2 Productivities Estimated with Prediction Model 2.

Scheme S1. De Novo-Designed Ligands Evaluated with Prediction Model 2.[a]

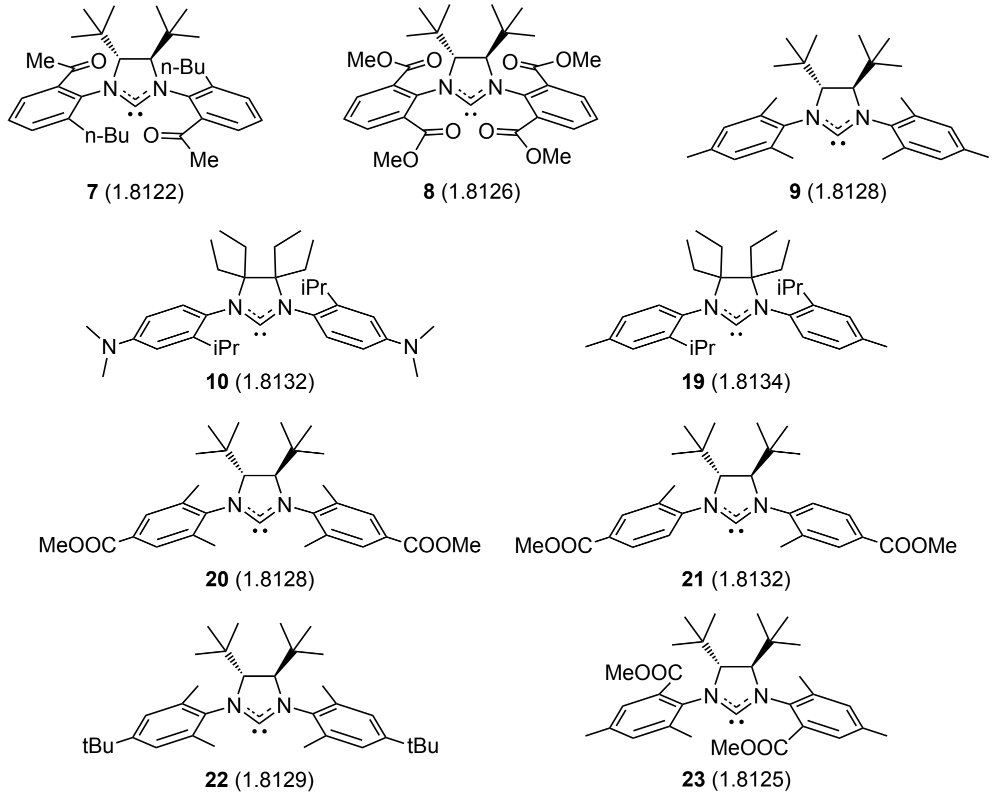

[a]The length of the $Ru=CH_2$ bond in the DFT model of **Ru5** generated in the evolutionary experiments is reported in parenthesis.

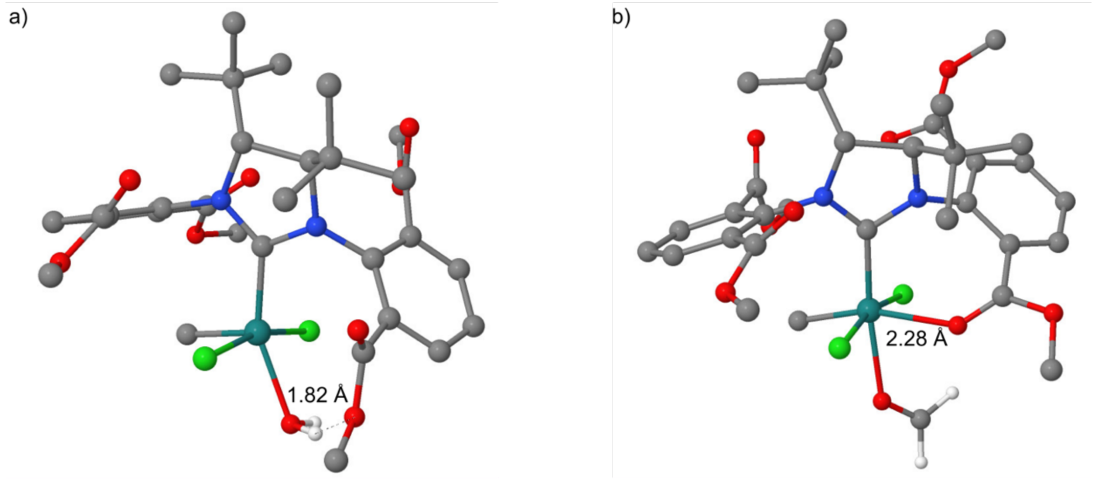

Figure S9. Molecular models Ru8_8_H2O_PR2 (a) and Ru8_8_CH2O_PR2 (b) showing effects that are not considered in *Prediction Model 2* and thus invalidate its prediction. Namely, the H-bond and metal-coordination resulting from the presence of carbonyl-containing moieties in the ortho position of the aromatic N-substituents on the NHC. All hydrogen atoms excluding those of water and formaldehyde are omitted for clarity. The labels report the distance H_{water}–O_{ester} (a) and Ru–$O_{carbonyl}$ (b).

Table S4. Calculated Energies, Enthalpies, and Productivities Computed with Prediction Model 2.

Molecular Model ID	$E_{OLYP,X}$ [a.u.]	$E_{B3LYP,X}$ [a.u.]	$H^{corr}_{OLYP,X}$ [a.u.]	Productivity [a.u.][a]	Productivity [kcal/mol]
CH2O_PR2	-114.454180	-114.532686	0.029867		
water_PR2	-76.390557	-76.452858	0.024272		
PMe3_PR2	-126.153869	-126.284132	0.120591		
ethylene_PR2	-78.543689	-78.606264	0.054557		
Ru8_7_CH2O_PR2	-1900.531803	-1901.786157	0.889684	ND[b]	ND[b]
Ru8_7_H2O_PR2	-1862.482540	-1863.713553	0.884979		
Ru8_7_PMe3_PR2	-1912.243660	-1913.563421	0.980586		
Ru9_7_PR2	-1864.635750	-1865.877078	0.917152		
Ru8_8_CH2O_PR2	-2192.157441	-2193.554868	0.754621	ND[b]	ND[b]
Ru8_8_H2O_PR2	-2154.123331	-2155.493829	0.750691		
Ru8_8_PMe3_PR2	-2203.890422	-2205.351915	0.846548		
Ru9_8_PR2	-2156.285432	-2157.664025	0.782672		
Ru8_9_CH2O_PR2	-1516.838832	-1517.822554	0.748681	0.008614	5.4
Ru8_9_H2O_PR2	-1478.790898	-1479.751078	0.743657		
Ru8_9_PMe3_PR2	-1528.553362	-1529.602306	0.839427		
Ru9_9_PR2	-1480.950639	-1481.918885	0.775488		
Ru8_10_CH2O_PR2	-1784.586857	-1785.781612	0.904079	0.012825	8.1
Ru8_10_H2O_PR2	-1746.541315	-1747.715263	0.899251		
Ru8_10_PMe3_PR2	-1796.309781	-1797.568956	0.995781		
Ru9_10_PR2	-1748.707818	-1749.886733	0.931610		
Ru8_19_CH2O_PR2	-1595.397579	-1596.445906	0.808959	0.011154	7.0
Ru8_19_H2O_PR2	-1557.351925	-1558.379127	0.803981		
Ru8_19_PMe3_PR2	-1607.120591	-1608.233027	0.899320		
Ru9_19_PR2	-1559.517811	-1560.549801	0.836655		
Ru8_20_CH2O_PR2	-1893.820587	-1895.038605	0.782242	0.008264	5.2
Ru8_20_H2O_PR2	-1855.773088	-1856.966789	0.777157		
Ru8_20_PMe3_PR2	-1905.536336	-1906.819186	0.873281		
Ru9_20_PR2	-1857.932567	-1859.135013	0.809391		
Ru8_21_CH2O_PR2	-1815.271325	-1816.419308	0.722840	0.006957	4.4
Ru8_21_H2O_PR2	-1777.221667	-1778.345692	0.717934		
Ru8_21_PMe3_PR2	-1826.986871	-1828.198750	0.813957		
Ru9_21_PR2	-1779.381477	-1780.513278	0.749923		
Ru8_22_CH2O_PR2	-1752.548901	-1753.733640	0.925045	0.00890	5.6
Ru8_22_H2O_PR2	-1714.500953	-1715.662198	0.920049		
Ru8_22_PMe3_PR2	-1764.263025	-1765.512881	1.016191		
Ru9_22_PR2	-1716.660752	-1717.830246	0.952143		

Ru8_23_CH2O_PR2	-1893.805117	-1895.027065	0.779191	ND[b]	ND[b]
Ru8_23_H2O_PR2	-1855.755098	-1856.952321	0.775608		
Ru8_23_PMe3_PR2	-1905.517315	-1906.803742	0.872149		
Ru9_23_PR2	-1857.911931	-1859.117080	0.808822		

[a]Productivity calculated according to Equation 1. [b]Not determinable due to effects as the H-bonds and over-coordination of the metal center (Figure S9) that are not accounted for by *Predicion Model 2*.

S2.2.3 Reaction Energy Profiles for Precatalyst 17 and 18

Scheme S2. Definition of Species Modelled to Determine the Reaction Energy Profiles for Precatalysts 17 and 18, which bear ligand 11 and 9, respectively.

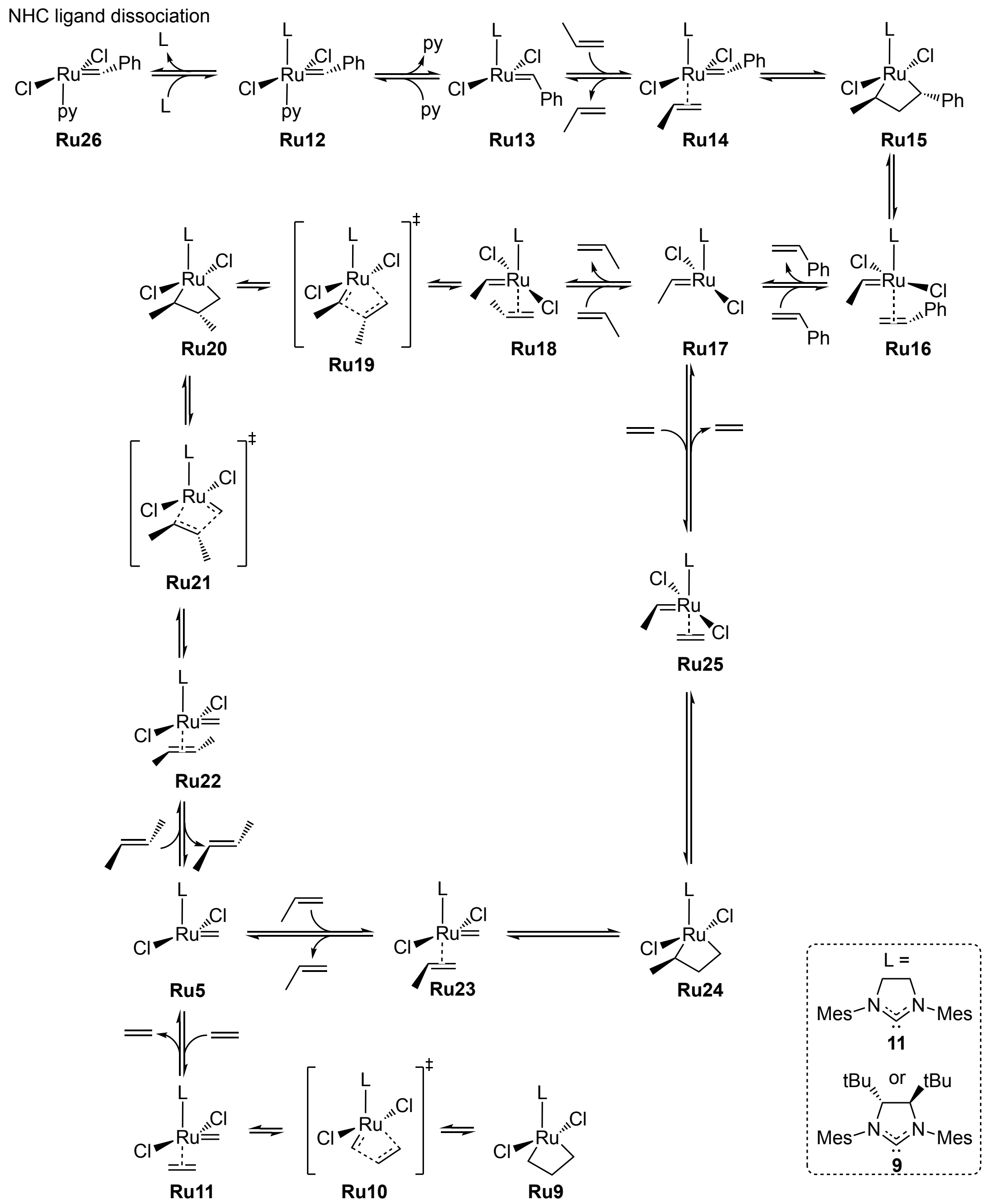

Table S5. Calculated Energies and Standard-State Gibbs Free Energies.

Molecular Model ID	E_{PBE} [a.u.]	$G^{298.15K}_{PBE\ qh}$ [a.u.]	$E^{C_6H_6}_{PBE\text{-}D3M(BJ)}$ [a.u.]	$G^{C_6H_6\ 298.15K\ [1M]}_{PBE\text{-}D3M(BJ)}$ [a.u.]	$\Delta G^{C_6H_6\ 298.15K\ [1M]}_{PBE\text{-}D3M(BJ)}$ [a] [kcal/mol]
pyridine	-247.997902	0.058677	-248.088944	-248.0272513	-
styrene	-309.273876	0.098810	-309.381907	-309.2800813	-
propene	-117.740843	0.052071	-117.790505	-117.7354179	-
ethylene	-78.473816	0.028509	-78.507080	-78.47555534	-
Ru12_9	-2771.334223	0.750194	-2771.968606	-2771.215395	0.0
Ru12_11	-2457.245853	0.538853	-2457.769495	-2457.227626	0.0
Ru13_9	-2523.278446	0.666245	-2523.838040	-2523.168779	12.2
Ru13_11	-2209.195907	0.454065	-2209.642729	-2209.185648	9.2
Ru19_9	-2449.507544	0.692843	-2450.054583	-2449.358724	12.7
Ru19_11	-2135.423191	0.482333	-2135.859619	-2135.374270	10.6
Ru20_9	-2449.526370	0.696544	-2450.074318	-2449.374759	2.6
Ru20_11	-2135.434734	0.485024	-2135.870504	-2135.382464	5.4
Ru21_9	-2449.502063	0.692490	-2450.050754	-2449.355248	14.8
Ru21_11	-2135.415223	0.482121	-2135.851523	--2135.366386	15.5
Ru9_9	-2370.997507	0.644247	-2371.518878	-2370.871616	-7.8
Ru9_11	-2056.904716	0.432855	-2057.314437	-2056.878566	-4.5
Ru10_9A[b]	-2370.970176	0.641854	-2371.491081	-2370.846209	8.1
Ru10_9B[b]	-2370.969556	0.641698	-2371.489849	-2370.845133	8.8
Ru10_11	-2056.880967	0.429541	-2057.289367	-2056.856807	9.2
9	-1238.382360	0.567584	-1238.815760	-1238.245159	-
11	-924.286350	0.356993	-924.612070	-924.252061	-
Ru26	-1532.844617	0.151373	-1533.068102	-1532.913713	0.0[c]
Ru26 + 9					-35.5[c]
Ru26 + 11					-38.8[c]

[a]Unless otherwise specified, values are calculated usen the corresponding model of **Ru12** as reference, namely **Ru12_9** for all species bearing ligand **9**, and **Ru12_11** for all species bearing ligand **11**. [b]Alternative configurations. [c]Relative to **Ru26**.

S3 Experimental Part

S3.1 Experimental Details

All reactions were performed under argon atmosphere inside a glovebox. Toluene and tetrahydrofuran were purified using an MBraun solvent purification system ("Grubbs' column") and stored over activated molecular sieves (4 Å). Anhydrous pentane was purchased from Sigma-Aldrich and used as received. $CDCl_3$ was dried over CaH_2 and distilled before use, while anhydrous C_6D_6 was purchased from Sigma-Aldrich and degassed before use. 1-octene (Sigma-Aldrich) was purified by passage through a column of activated basic alumina and stored over activated Selexsorb® CD in the glovebox freezer for 1 week before use. The imidazolium salt **9·HCl** was purchased from Santai Labs, Inc. and used as received. The ruthenium compounds **16**[48] and **18**[49] were prepared according to literature procedures. All the other chemicals were purchased from Sigma-Aldrich and used as received.

NMR spectra were recorded on Bruker Biospin AV 500, AVANCE NEO 600, and AV III HD 850 spectrometers. The chemical shifts are reported relative to the residual solvent peaks.[50] Elemental analyses were performed using an Elementar Vario EL III analyzer.

S3.2 Preparation of Ruthenium Complex 17

Scheme S3. Preparation of Complex 17.

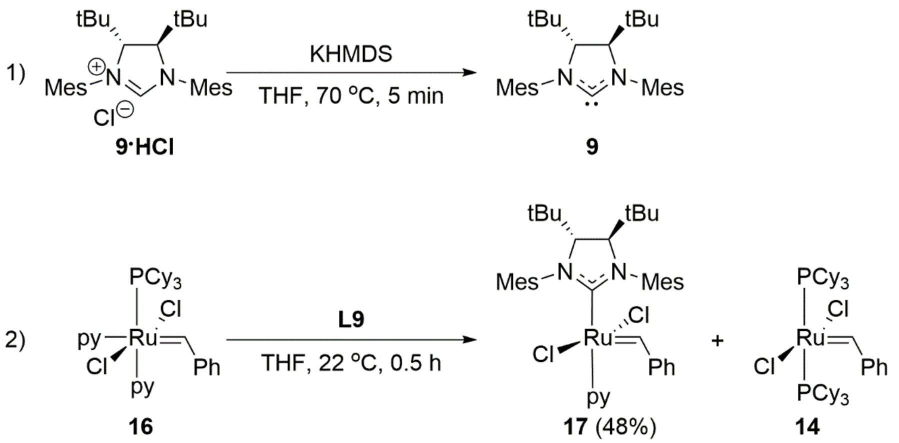

In a glovebox, a 25 mL vial, equipped with a magnetic stirring bar and a screw cap, was charged with the imidazolium salt **9·HCl** (25 mg, 0.055 mmol), potassium bis(trimethylsilyl)amide (13.2 mg, 0.066 mmol), and tetrahydrofuran (4 mL). The suspension was heated at 70° C for 5 minutes, then filtered through a glass-fiber filter paper, and the filtrate was transferred to a 2 mL THF solution of **16** (36.6 mg, 0.052 mmol). The mixture was stirred at room temperature for 30 minutes. During the first five minutes the color of the solution changed from bright green to brown-orange. The solvent was reduced under reduced pressure to about one third of the initial amount, then pentane (10 mL) was added dropwise under magnetic stirring. The addition of pentane caused the separation of the title compound as a green solid, which was isolated by vacuum filtration, washed three times with pentane, transferred in a vial, and dried under reduced pressure. Isolated weight: 19 mg (48 %). ^1H NMR (850.13 MHz, C_6D_6, 298 K): δ = 19.32 (s, 1H), 8.41 (m, 2H), 8.20 (br, 2H), 7.20 (t, J = 7.2 Hz, 1H), 6.96 (br, 2H), 6.94 (br t, J = 7.8 Hz, 2H), 6.91 (br s, 1H), 6.56 (tt, J = 7.5, 1.6 Hz, 1H), 6.51 (br s, 1H), 6.25 (m, 2H), 4.46 (d, J = 6.6 Hz, 1H), 4.15 (d, J = 6.6 Hz, 1H), 3.48 (s, 3H), 3.08 (s, 3H), 2.47 (s, 3H), 2.221 (s, 3H), 2.216 (s, 3H), 1.86 (s, 3H),1.23 (s, 9H), 0.77 (s, 9H). 13C{1H} NMR (213.77 MHz, C_6D_6, 298 K): δ = 319.05, 222.81, 154.22, 151.55, 141.53, 140.68, 140.03, 139.99, 138.06, 137.60, 136.82, 136.32, 136.25, 131.88, 131.84, 131.71, 131.04, 130.72, 130.52, 130.24, 129.33, 128.50, 123.29, 74.86, 73.65, 36.91, 35.71, 30.33, 29.49, 24.93, 23.82, 22.55, 22.34, 20.92, 20.87. Elemental analysis, calculated for $C_{41}H_{53}Cl_2N_3Ru$: C, 64.81, H, 7.03, N, 5.53; found: C, 64.71, H, 6.79, N, 5.25.

S3.3 Self-Metathesis of Neat 1-Octene with 100 ppm of Catalyst at Room Temperature (Figure 2, Main Paper)

In a glove box, a 25 mL vial equipped with a magnetic stirring bar and a screw cap was charged with 2954 mg (13.160 mmol) of 1-octene, 59.5 mg (0.366 mmol) of hexamethylbenzene (internal standard) and the catalyst (0.00132 mmol, 0.01 mol %). The vial was closed, and the reaction mixture was stirred at room temperature (22 °C) for 24 hours. Samples were taken at 5, 10, 15, 30, 60, 120, 240, and 1440 minutes. Each sample was quenched with an excess of ethyl vinyl ether (EVE) and stored in the glovebox freezer at -35 °C before ^1H NMR analysis. Determination of conversions and yields were done by quantitative ^1H NMR according to literature procedures.[51,52]

S3.4 Self-Metathesis of Neat 1-Octene with 1 ppm of Catalyst at 60 °C

In a glove box, a 4 mL vial equipped with a magnetic stirring bar and a screw cap was charged with 10 mg of stock solution, containing 0.0026 µmol of the catalyst. The solvent was removed by reduced pressure and then (2.6 mmol) of 1-octene were added. The vial was closed, and the reaction mixture was stirred at 60 °C for 1 hour. Determination of conversions and yields were done by quantitative ^{1}H NMR according to literature procedures.[51,52]

S3.5 NMR Spectra of 17

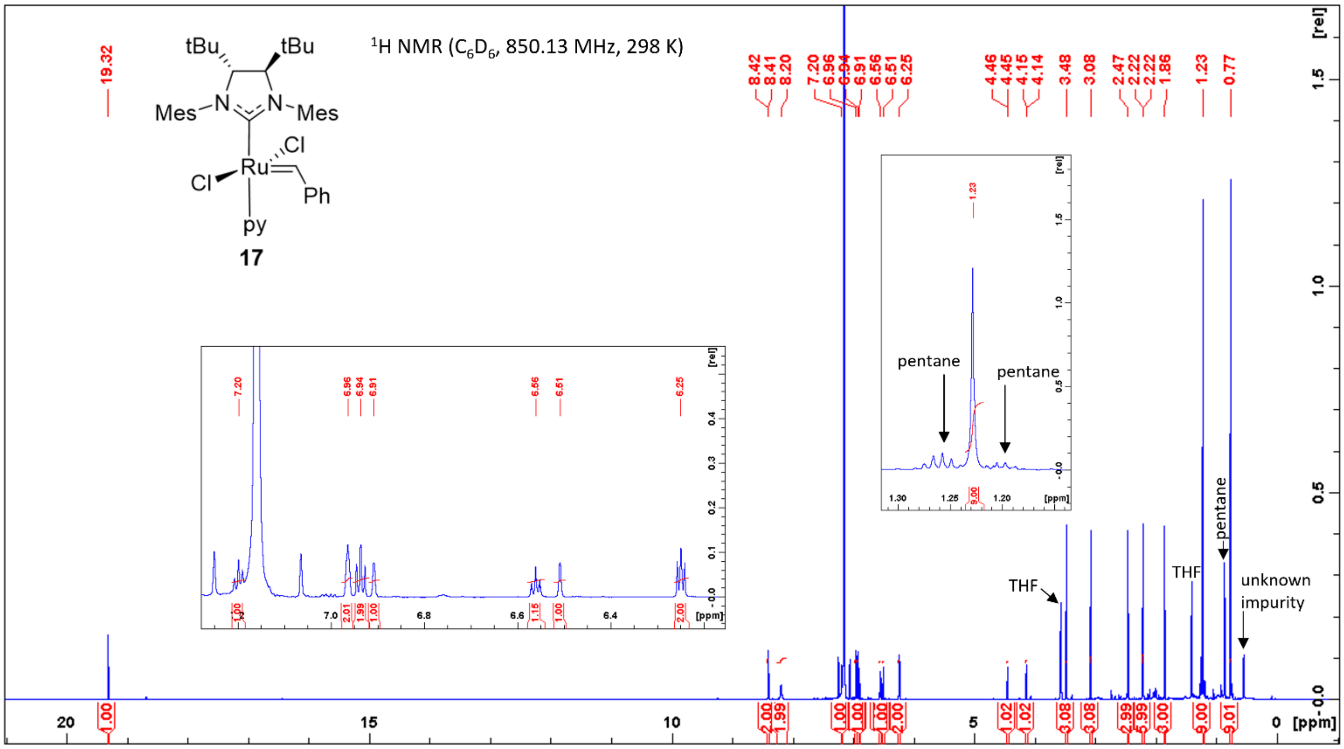

Figure S10. ^{1}H NMR spectrum of **17**.

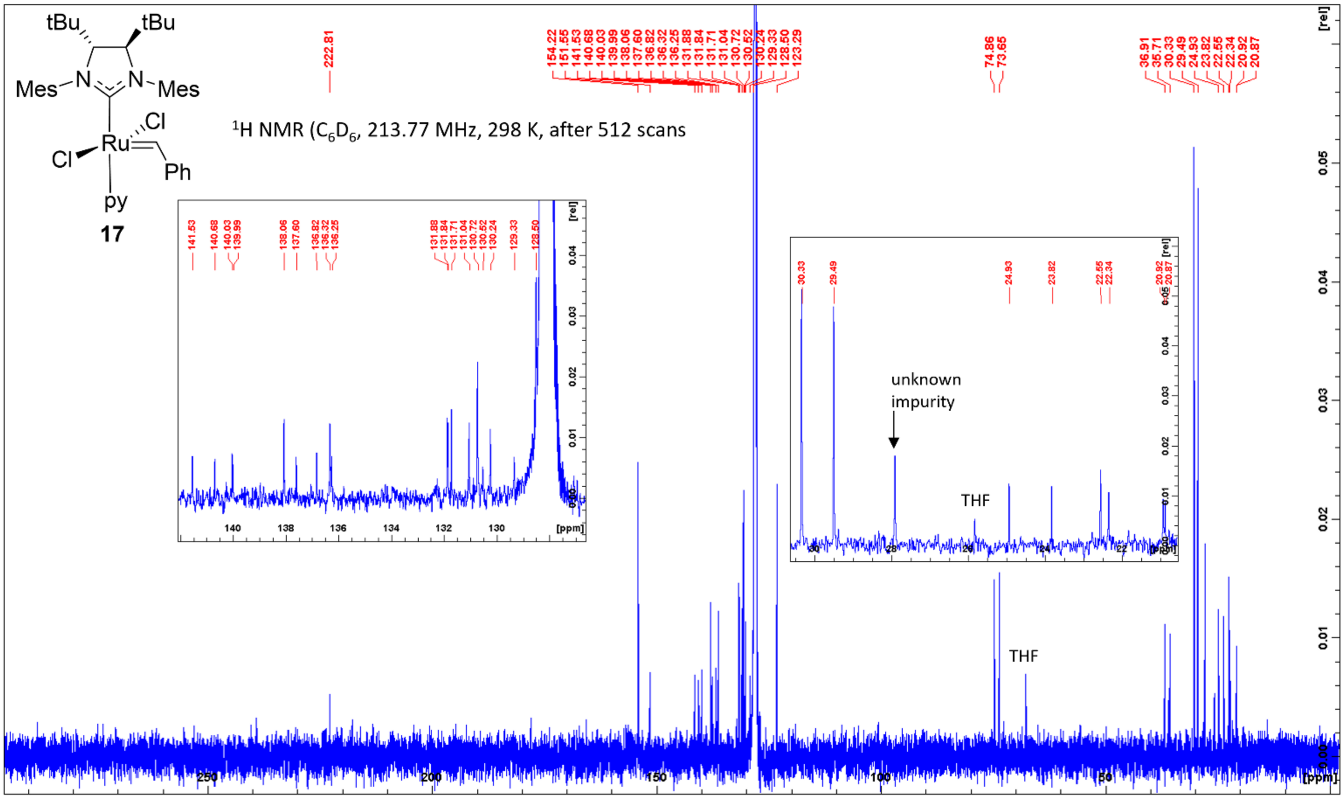

Figure S11. ^{13}C NMR spectrum of **17**, recorded after 512 scans.

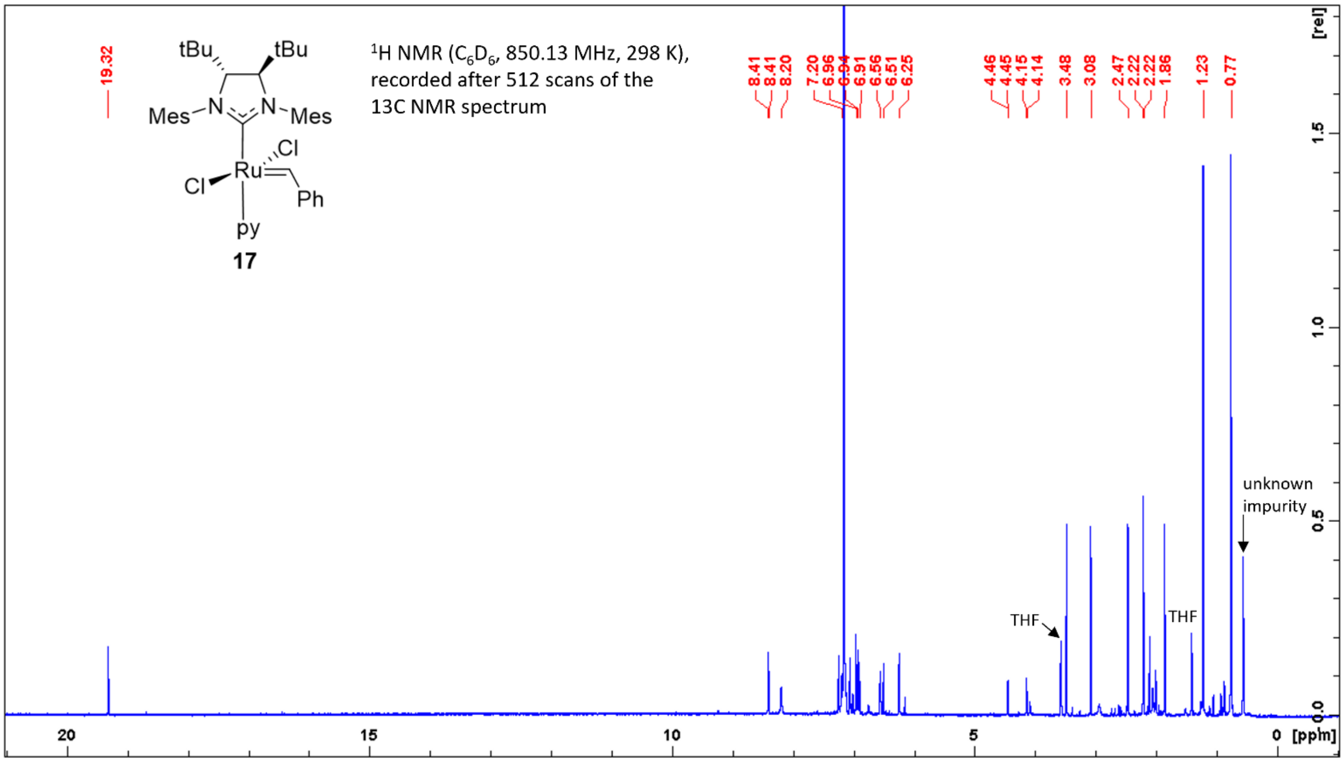

Figure S12. ^{1}H NMR spectrum of **17** (1 scan), recorded after the first 512 scan of the ^{13}C NMR spectrum.

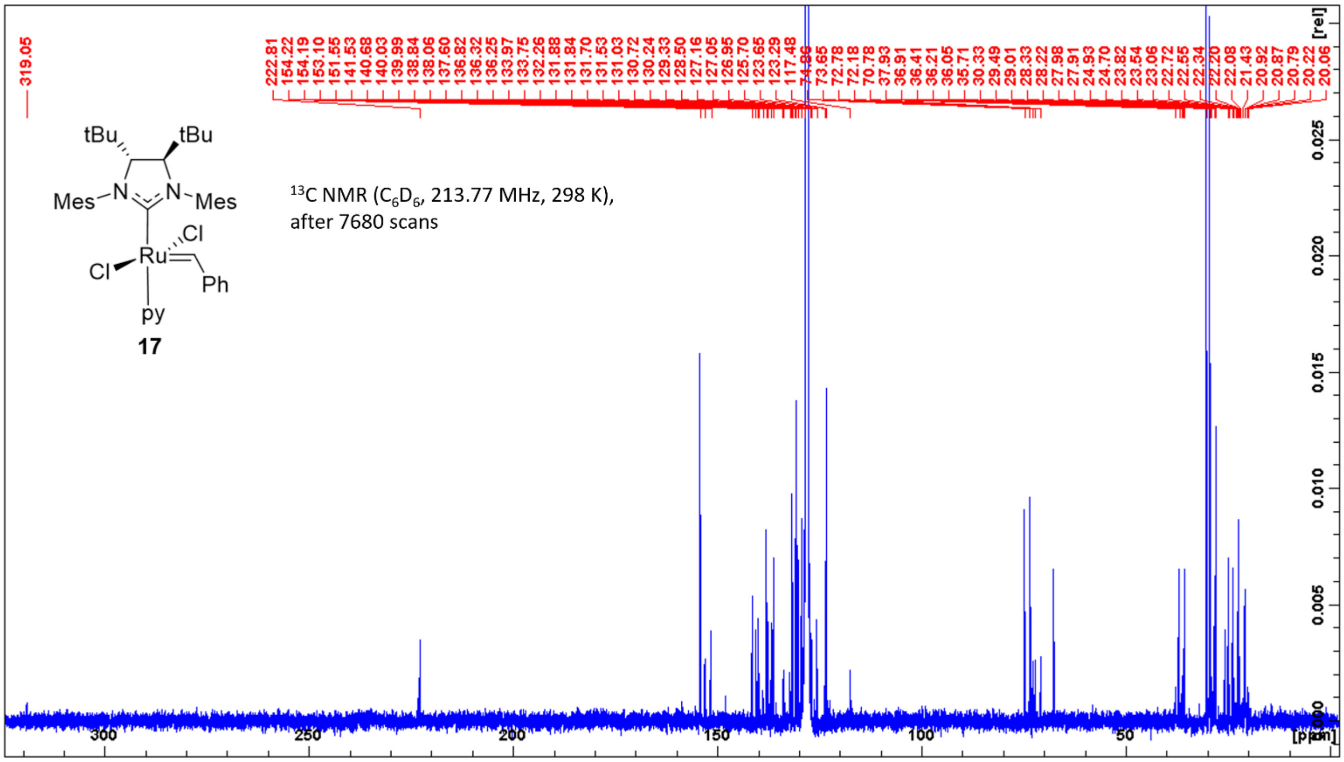

Figure S13. ^{13}C NMR spectrum of **17**, recorded after 7680 scans.

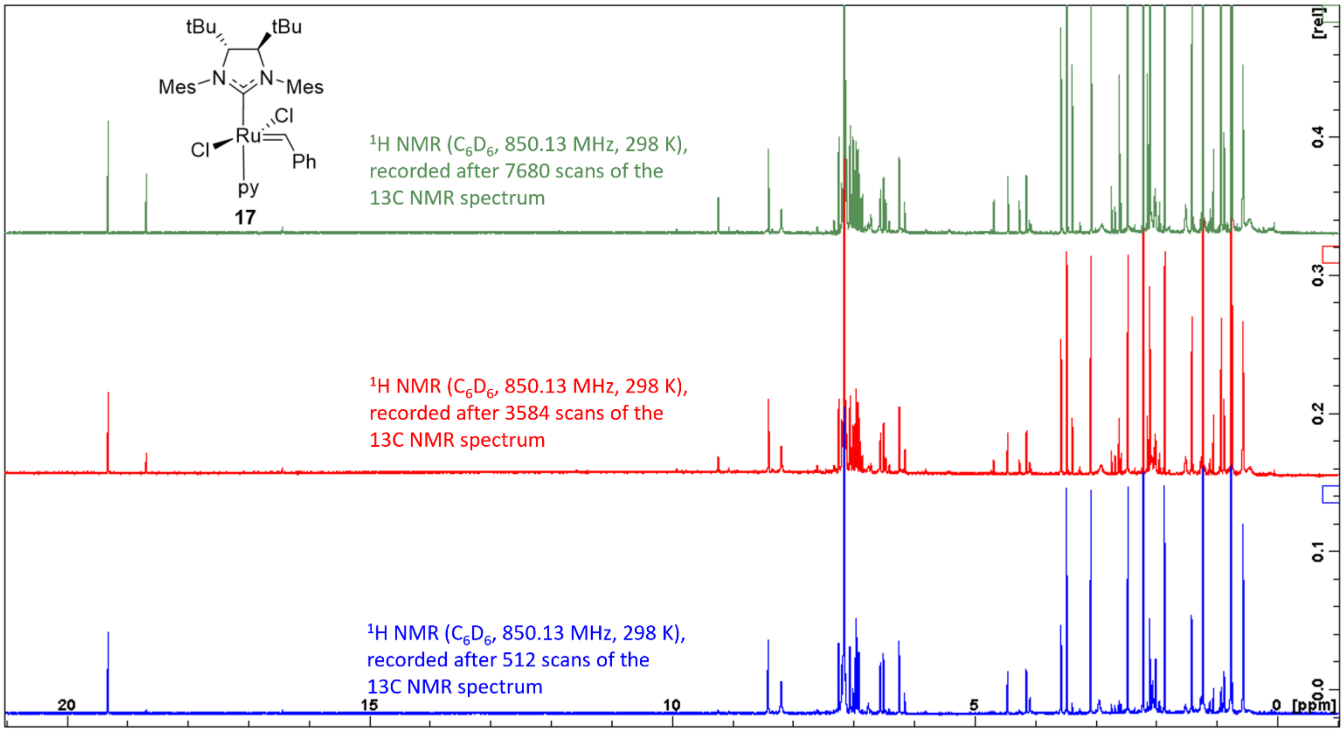

Figure S14. Stacked ^{1}H NMR spectrum of **17** (1 scan) showing the formation of a new alkylidene species during the recording of the ^{13}C NMR spectrum.

S4 References

(1) Adlhart, C.; Chen, P. Mechanism and Activity of Ruthenium Olefin Metathesis Catalysts: The Role of Ligands and Substrates from a Theoretical Perspective. *J. Am. Chem. Soc.* **2004**, *126*, 3496–3510. https://doi.org/10.1021/ja0305757.

(2) Occhipinti, G.; Bjørsvik, H.-R.; Jensen, V. R. Quantitative Structure−Activity Relationships of Ruthenium Catalysts for Olefin Metathesis. *J. Am. Chem. Soc.* **2006**, *128*, 6952–6964. https://doi.org/10.1021/ja060832i.

(3) Marenich, A. V.; Cramer, C. J.; Truhlar, D. G. Universal Solvation Model Based on Solute Electron Density and on a Continuum Model of the Solvent Defined by the Bulk Dielectric Constant and Atomic Surface Tensions. *J. Phys. Chem. B* **2009**, *113*, 6378–6396. https://doi.org/10.1021/jp810292n.

(4) Foscato, M.; Venkatraman, V.; Occhipinti, G.; Alsberg, B. K.; Jensen, V. R. Automated Building of Organometallic Complexes from 3D Fragments. *J. Chem. Inf. Model.* **2014**, *54*, 1919–1931. https://doi.org/10.1021/ci5003153.

(5) Ponder, J. W. TINKER: Software Tools for Molecular Design, 4.2, Saint Louis, MO. **2004**.

(6) Rappe, A. K.; Casewit, C. J.; Colwell, K. S.; Goddard, W. A.; Skiff, W. M. UFF, a Full Periodic Table Force Field for Molecular Mechanics and Molecular Dynamics Simulations. *J. Am. Chem. Soc.* **1992**, *114*, 10024–10035. https://doi.org/10.1021/ja00051a040.

(7) M. J. Frisch; G. W. Trucks; H. B. Schlegel; G. E. Scuseria; M. A. Robb; J. R. Cheeseman; G. Scalmani; V. Barone; B. Mennucci; G. A. Petersson; H. Nakatsuji; M. Caricato; X. Li; H. P. Hratchian; A. F. Izmaylov; J. Bloino; G. Zheng; J. L. Sonnenberg; M. Hada; M. Ehara; K. Toyota; R. Fukuda; J. Hasegawa; M. Ishida; T. Nakajima; Y. Honda; O. Kitao; H. Nakai; T. Vreven; J. A. Montgomery, Jr.; J. E. Peralta; F. Ogliaro; M. Bearpark; J. J. Heyd; E. Brothers; K. N. Kudin; V. N. Staroverov; R. Kobayashi; J. Normand; K. Raghavachari; A. Rendell; J. C. Burant; S. S. Iyengar; J. Tomasi; M. Cossi; N. Rega; J. M. Millam; M. Klene; J. E. Knox; J. B. Cross; V. Bakken; C. Adamo; J. Jaramillo; R. Gomperts; R. E. Stratmann; O. Yazyev; A. J. Austin; R. Cammi; C. Pomelli; J. W. Ochterski; R. L. Martin; K. Morokuma; V. G. Zakrzewski; G. A. Voth; P. Salvador; J. J. Dannenberg; S. Dapprich; A. D. Daniels; Ö. Farkas; J. B. Foresman; J. V. Ortiz; J. Cioslowski; Fox, D. J. Gaussian 09, 2009.

(8) Frisch, M. J.; Trucks, G. W.; Schlegel, H. B.; Scuseria, G. E.; Robb, M. A.; Cheeseman, J. R.; Scalmani, G.; Barone, V.; Petersson, G. A.; Nakatsuji, H.; Li, X.; Caricato, M.; Marenich, A. V.; Bloino, J.; Janesko, B. G.; Gomperts, R.; Mennucci, B.; Hratchian, H. P.; Ortiz, J. V.; Izmaylov, A. F.; Sonnenberg, J. L.; Williams-Young, D.; Ding, F.; Lipparini, F.; Egidi, F.; Goings, J.; Peng, B.; Petrone, A.; Henderson, T.; Ranasinghe, D.; Zakrzewski, V. G.; J. Gao, N. R.; Zheng, G.; Liang, W.; Hada, M.; Ehara, M.; Toyota, K.; Fukuda, R.; Hasegawa, J.; Ishida, M.; Nakajima, T.; Honda, Y.; Kitao, O.; Nakai, H.; Vreven, T.; Throssell, K.; J. A. Montgomery, Jr.; Peralta, J. E.; Ogliaro, F.; Bearpark, M. J.; Heyd, J. J.; Brothers, E. N.; Kudin, K. N.; Staroverov, V. N.; Keith, T. A.; Kobayashi, R.; Normand, J.; Raghavachari, K.; Rendell, A. P.; Burant, J. C.; Iyengar, S. S.; Tomasi, J.; Cossi, M.; Millam, J. M.; Klene, M.; Adamo, C.; Cammi, R.; Ochterski, J. W.; Martin, R. L.; Morokuma, K.; Farkas, O.; Foresman, J. B.; Fox, D. J. Gaussian 16, 2016.

(9) Handy, N. C.; Cohen, A. J. Left-Right Correlation Energy. *Mol. Phys.* **2001**, *99*, 403–412. https://doi.org/10.1080/00268970010018431.

(10) Hoe, W. M.; Cohen, A. J.; Handy, N. C. Assessment of a New Local Exchange Functional OPTX. *Chem. Phys. Lett.* **2001**, *341*, 319–328. https://doi.org/10.1016/S0009-2614(01)00581-4.

(11) Lee, C. T.; Yang, W. T.; Parr, R. G. Development of the Colle-Salvetti Correlation-Energy Formula Into A Functional of the Electron-Density. *Phys. Rev. B* **1988**, *37*, 785–789. https://doi.org/10.1103/PhysRevB.37.785.

(12) Miehlich, B.; Savin, A.; Stoll, H.; Preuss, H. Results Obtained With the Correlation-Energy Density Functionals of Becke and Lee, Yang, and Parr. *Chem. Phys. Lett.* **1989**, *157*, 200–206. https://doi.org/10.1016/0009-2614(89)87234-3.
(13) Hay, P. J.; Wadt, W. R. Abinitio Effective Core Potentials for Molecular Calculations - Potentials for the Transition-Metal Atoms Sc to Hg. *J. Chem. Phys.* **1985**, *82*, 270–283.
(14) Hay, P. J.; Wadt, W. R. Ab Initio Effective Core Potentials for Molecular Calculations. Potentials for Main Group Elements Na to Bi. *J. Chem. Phys.* **1985**, *82*, 284–298.
(15) Hay, P. J.; Wadt, W. R. Abinitio Effective Core Potentials for Molecular Calculations - Potentials for K to Au Including the Outermost Core Orbitals. *J. Chem. Phys.* **1985**, *82*, 299–310.
(16) O'Boyle, N. M.; Banck, M.; James, C. A.; Morley, C.; Vandermeersch, T.; Hutchison, G. R. Open Babel: An Open Chemical Toolbox. *J. Cheminformatics* **2011**, *3*. https://doi.org/10.1186/1758-2946-3-33.
(17) Wavefunction Inc. Spartan '08, 2008.
(18) Stewart, J. J. P. Optimization of Parameters for Semiempirical Methods IV: Extension of MNDO, AM1, and PM3 to More Main Group Elements. *J. Mol. Model.* **2004**, *10*, 155–164. https://doi.org/10.1007/s00894-004-0183-z.
(19) Becke, A. D. Density-functional Thermochemistry. III. The Role of Exact Exchange. *J. Chem. Phys.* **1993**, *98*, 5648–5652. https://doi.org/10.1063/1.464913.
(20) Hay, P. J.; Wadt, W. R. Ab Initio Effective Core Potentials for Molecular Calculations. Potentials for K to Au Including the Outermost Core Orbitals. *J. Chem. Phys.* **1985**, *82*, 299–310.
(21) Check, C. E.; Faust, T. O.; Bailey, J. M.; Wright, B. J.; Gilbert, T. M.; Sunderlin, L. S. Addition of Polarization and Diffuse Functions to the LANL2DZ Basis Set for P-Block Elements. *J. Phys. Chem. A* **2001**, *105*, 8111–8116. https://doi.org/10.1021/jp011945l.
(22) Álvarez-Moreno, M.; de Graaf, C.; López, N.; Maseras, F.; Poblet, J. M.; Bo, C. Managing the Computational Chemistry Big Data Problem: The ioChem-BD Platform. *J. Chem. Inf. Model.* **2015**, *55*, 95–103. https://doi.org/10.1021/ci500593j.
(23) Bo, C.; Maseras, F.; López, N. The Role of Computational Results Databases in Accelerating the Discovery of Catalysts. *Nat. Catal.* **2018**, *1*, 809–810. https://doi.org/10.1038/s41929-018-0176-4.
(24) Spartan'18, 2018.
(25) Halgren, T. A. Merck Molecular Force Field .1. Basis, Form, Scope, Parameterization, and Performance of MMFF94. *J. Comput. Chem.* **1996**, *17*, 490–519.
(26) Stewart, J. J. P. Optimization of Parameters for Semiempirical Methods V: Modification of NDDO Approximations and Application to 70 Elements. *J. Mol. Model.* **2007**, *13*, 1173–1213. https://doi.org/10.1007/s00894-007-0233-4.
(27) Frisch, M. J.; Trucks, G. W.; Schlegel, H. B.; Scuseria, G. E.; Robb, M. A.; Cheeseman, J. R.; Scalmani, G.; Barone, V.; Petersson, G. A.; Nakatsuji, H.; Li, X.; Caricato, M.; Marenich, A. V.; Bloino, J.; Janesko, B. G.; Gomperts, R.; Mennucci, B.; Hratchian, H. P.; Ortiz, J. V.; Izmaylov, A. F.; Sonnenberg, J. L.; Williams-Young, D.; Ding, F.; Lipparini, F.; Egidi, F.; Goings, J.; Peng, B.; Petrone, A.; Henderson, T.; Ranasinghe, D.; Zakrzewski, V. G.; Gao, J.; Rega, N.; Zheng, G.; Liang, W.; Hada, M.; Ehara, M.; Toyota, K.; Fukuda, R.; Hasegawa, J.; Ishida, M.; Nakajima, T.; Honda, Y.; Kitao, O.; Nakai, H.; Vreven, T.; Throssell, K.; Montgomery, J. A. Jr.; Peralta, J. E.; Ogliaro, F.; Bearpark, M. J.; Heyd, J. J.; Brothers, E. N.; Kudin, K. N.; Staroverov, V. N.; Keith, T. A.; Kobayashi, R.; Normand, J.; Raghavachari, K.; Rendell, A. P.; Burant, J. C.; Iyengar, S. S.; Tomasi, J.; Cossi, M.; Millam, J. M.; Klene, M.; Adamo, C.; Cammi, R.; Ochterski, J. W.; Martin, R. L.; Morokuma, K.; Farkas, O.; Foresman, J. B.; Fox, D. J. Gaussian 16 Revision C.01, 2016.
(28) Perdew, J.; Burke, K.; Ernzerhof, M. Generalized Gradient Approximation Made Simple [Phys. Rev. Lett. 77, 3865 (1996)]. *Phys. Rev. Lett.* **1997**, *78*, 1396. https://doi.org/10.1103/PhysRevLett.78.1396.

(29) Perdew, J.; Burke, K.; Ernzerhof, M. Generalized Gradient Approximation Made Simple. *Phys. Rev. Lett.* **1996**, *77*, 3865–3868. https://doi.org/10.1103/PhysRevLett.77.3865.
(30) Dunning, T. H. Gaussian Basis Sets for Use in Correlated Molecular Calculations. I. The Atoms Boron through Neon and Hydrogen. *J. Chem. Phys.* **1989**, *90*, 1007–1023. https://doi.org/10.1063/1.456153.
(31) Woon, D. E.; Dunning, T. H. Gaussian Basis Sets for Use in Correlated Molecular Calculations. III. The Atoms Aluminum through Argon. *J. Chem. Phys.* **1993**, *98*, 1358–1371. https://doi.org/10.1063/1.464303.
(32) Feller, D. The Role of Databases in Support of Computational Chemistry Calculations. *J. Comput. Chem.* **1996**, *17*, 1571–1586. https://doi.org/10.1002/(SICI)1096-987X(199610)17:13<1571::AID-JCC9>3.0.CO;2-P.
(33) Schuchardt, K. L.; Didier, B. T.; Elsethagen, T.; Sun, L. S.; Gurumoorthi, V.; Chase, J.; Li, J.; Windus, T. L. Basis Set Exchange: A Community Database for Computational Sciences. *J. Chem. Inf. Model.* **2007**, *47*, 1045–1052. https://doi.org/10.1021/ci600510j.
(34) Peterson, K. A.; Figgen, D.; Dolg, M.; Stoll, H. Energy-Consistent Relativistic Pseudopotentials and Correlation Consistent Basis Sets for the 4d Elements Y–Pd. *J. Chem. Phys.* **2007**, *126*, 124101. https://doi.org/10.1063/1.2647019.
(35) Ribeiro, R. F.; Marenich, A. V.; Cramer, C. J.; Truhlar, D. G. Use of Solution-Phase Vibrational Frequencies in Continuum Models for the Free Energy of Solvation. *J. Phys. Chem. B* **2011**, *115*, 14556–14562. https://doi.org/10.1021/jp205508z.
(36) Grimme, S.; Ehrlich, S.; Goerigk, L. Effect of the Damping Function in Dispersion Corrected Density Functional Theory. *J. Comput. Chem.* **2011**, *32*, 1456–1465. https://doi.org/10.1002/jcc.21759.
(37) Smith, D. G. A.; Burns, L. A.; Patkowski, K.; Sherrill, C. D. Revised Damping Parameters for the D3 Dispersion Correction to Density Functional Theory. *J. Phys. Chem. Lett.* **2016**, *7*, 2197–2203. https://doi.org/10.1021/acs.jpclett.6b00780.
(38) Energy-consistent Pseudopotentials of the Stuttgart/Cologne Group http://www.tc.uni-koeln.de/PP/clickpse.en.html.
(39) Kendall, R. A.; Dunning, T. H.; Harrison, R. J. Electron Affinities of the First-Row Atoms Revisited. Systematic Basis Sets and Wave Functions. *J Chem Phys* **1992**, *96*, 6796–6806. https://doi.org/10.1063/1.462569.
(40) Cossi, M.; Scalmani, G.; Rega, N.; Barone, V. New Developments in the Polarizable Continuum Model for Quantum Mechanical and Classical Calculations on Molecules in Solution. *J. Chem. Phys.* **2002**, *117*, 43–54. https://doi.org/10.1063/1.1480445.
(41) Scalmani, G.; Frisch, M. J. Continuous Surface Charge Polarizable Continuum Models of Solvation. I. General Formalism. **2010**, *132*, 114110. https://doi.org/10.1063/1.3359469.
(42) Tomasi, J.; Mennucci, B.; Cammi, R. Quantum Mechanical Continuum Solvation Models. *Chem. Rev.* **2005**, *105*, 2999–3093. https://doi.org/10.1021/cr9904009.
(43) Tomasi, J.; Persico, M. Molecular Interactions in Solution: An Overview of Methods Based on Continuous Distributions of the Solvent. *Chem Rev* **1994**, *94*, 2027–2094. https://doi.org/10.1021/cr00031a013.
(44) Tomasi, J. Thirty Years of Continuum Solvation Chemistry: A Review, and Prospects for the near Future. *Theor. Chem. Acc.* **2004**, *112*, 184–203.
(45) Cramer, C. J.; Truhlar, D. G. Density Functional Theory for Transition Metals and Transition Metal Chemistry. *Phys. Chem. Chem. Phys.* **2009**, *11*, 10757–10816. https://doi.org/10.1039/B907148B.
(46) Klamt, A.; Mennucci, B.; Tomasi, J.; Barone, V.; Curutchet, C.; Orozco, M.; Luque, F. J. On the Performance of Continuum Solvation Methods. A Comment on "Universal Approaches to Solvation Modeling." *Acc. Chem. Res.* **2009**, *42*, 489–492. https://doi.org/10.1021/ar800187p.

(47) Glendening, E. D.; Badenhoop, J. K.; Reed, A. E.; Carpenter, J. E.; Bohmann, J. A.; Morales, C. M.; Karafiloglou, P.; Landis, C. R.; Weinhold, F. NBO 7.0, 2018.
(48) Getty, K.; Delgado-Jaime, M. U.; Kennepohl, P. Assignment of Pre-Edge Features in the Ru K-Edge X-Ray Absorption Spectra of Organometallic Ruthenium Complexes. *Inorganica Chim. Acta* **2008**, *361*, 1059. https://doi.org/10.1016/j.ica.2007.07.029.
(49) Wolf, W. J.; Lin, T.-P.; Grubbs, R. H. Examining the Effects of Monomer and Catalyst Structure on the Mechanism of Ruthenium-Catalyzed Ring-Opening Metathesis Polymerization. *J. Am. Chem. Soc.* **2019**, *141*, 17796–17808. https://doi.org/10.1021/jacs.9b08835.
(50) Fulmer, G. R.; Miller, A. J. M.; Sherden, N. H.; Gottlieb, H. E.; Nudelman, A.; Stoltz, B. M.; Bercaw, J. E.; Goldberg, K. I. NMR Chemical Shifts of Trace Impurities: Common Laboratory Solvents, Organics, and Gases in Deuterated Solvents Relevant to the Organometallic Chemist. *Organometallics* **2010**, *29*, 2176–2179. https://doi.org/10.1021/om100106e.
(51) Keitz, B. K.; Endo, K.; Herbert, M. B.; Grubbs, R. H. Z-Selective Homodimerization of Terminal Olefins with a Ruthenium Metathesis Catalyst. *J. Am. Chem. Soc.* **2011**, *133*, 9686–9688. https://doi.org/10.1021/ja203488e.
(52) Occhipinti, G.; Hansen, F. R.; Törnroos, K. W.; Jensen, V. R. Simple and Highly Z-Selective Ruthenium-Based Olefin Metathesis Catalyst. *J. Am. Chem. Soc.* **2013**, *135*, 3331–3334. https://doi.org/10.1021/ja311505v.
